# Efficacy and safety of pseudolaric acid B against *Echinococcus multilocularis in vitro* and in a murine infection model

**DOI:** 10.3389/fmed.2025.1503472

**Published:** 2025-01-29

**Authors:** Zhuo-ma Dawa, Ting Zhai, Chuan-chuan Liu, Hai-ning Fan

**Affiliations:** ^1^Research Center for High Altitude Medicine, Key Laboratory of High Altitude Medicine (Ministry of Education), Key Laboratory of Application and Foundation for High Altitude Medicine Research in Qinghai Province (Qinghai-Utah Joint Research Key Lab for High Altitude Medicine), Laboratory for High Altitude Medicine of Qinghai Province, Qinghai University, Xining, China; ^2^Qinghai Province Key Laboratory of Echinococcosis, Xining, China; ^3^Qinghai University Affiliated Hospital, Xining, China

**Keywords:** pseudolaric acid B, *Echinococcus multilocularis*, protoscolex, lymphocyte, matrix metalloproteinases, collagen deposition

## Abstract

**Introduction:**

Alveolar echinococcosis (AE) is a chronic zoonotic disease caused by the larvae of the *Echinococcus multilocularis* (*E. multilocularis*). The current chemotherapy for AE relies on albendazole and mebendazole, which exhibit only parasitostatic rather than parasiticidal effects. Therefore, there is a need to find new anti-Echinococcosis drugs. Pseudolaric acid B (PAB) has been described to have strong antiparasitic effects. However, the in-depth mechanism by which PAB acts against *E. multilocularis* remains unclear.

**Methods:**

To evaluate the effect of a PAB intervention on protoscoleces, metacestode vesicles and germinal cells in *E. multilocularis in vitro*. In addition, the effects of PAB on T lymphocyte and collagen synthesis were evaluated after PAB administration in a mouse model.

**Results:**

Metacestode vesicles and germinal cells were successfully cultured, and specific genes were amplified via RT-PCR to identify the protoscoleces, vesicles, and germinal cells as the sources of *E. multilocularis*. *In vitro* studies have demonstrated that PAB exhibits dose- and concentration-dependent cytotoxicity against *E. multilocularis* protoscoleces. Scanning electron microscopy revealed that the microvilli structure of the protoscolex was destroyed and the rostellar hooks had fallen off. PAB induced. The release of PGI from the metacestode vesicles, leading to the structural destruction of the inner surfaces. PAB suppressed the proliferation of germinal cells. After PAB treatment, the serum and the host tissue surrounding the metacestodes IFN-*γ* levels were upregulated and the IL-4 and IL-10 levels was downregulated. After PAB treatment, the levels of CD4^+^ T lymphocytes increased and the levels of CD8^+^ T lymphocytes decreased in the host tissue surrounding the metacestodes and the spleen. The proportions of the Th1 and Th17 cell subpopulations were increased and the proportion of Th2 cell and Treg cell subpopulations was decreased in the host tissue surrounding the metacestodes. Additionally, collagen deposition was increased after PAB treatment. PAB suppressed the expression of matrix metalloproteinases (MMPs 1, 2, 3, 9, 13) and the activation of the PI3K/AKT signaling pathway in the host tissue surrounding the metacestodes.

**Conclusion:**

PAB has a significant killing effect on *E. multilocularis*, suggesting that it is a potential candidate for the treatment of AE.

## 1 Introduction

Alveolar echinococcosis (AE), caused by the larval stage of *Echinococcus multilocularis* (*E. multilocularis*), is a serious zoonotic parasitic disease predominantly affecting the northern hemisphere, with 91% of cases concentrated on the Tibetan Plateau in Western China (1). Humans are infected via the accidental ingestion of food and water contaminated with eggs (2). The AE targets the liver, where it forms infiltrative lesions that can spread to other organs, making treatment particularly challenging (3). Current therapeutic options for AE are limited and often inadequate. While surgical resection remains the primary treatment, it is frequently incomplete due to the infiltrative nature of the parasite, leading to high recurrence rates (4). Long-term benzimidazole therapy (albendazole and mebendazole) can suppress parasitic growth but cannot eliminate the infection and often causes adverse effects. Liver transplantation, though available for advanced cases, is complicated by severe immune rejection (5). These therapeutic limitations underscore the urgent need for novel, more effective drugs.

Pseudolaric acid B (PAB, C_23_H_28_O_8_), a diterpene with a structure that includes five-membered rings and a seven-membered ring, is a traditional Chinese medicine that has been used for treatment of fungal-infected dermatosis for many years (6, 7). Increasing evidences indicated that PAB showed various bioactivities with less cytotoxicity, including antimicrobial activity, antifertility activity, antiangiogenic activity, anticancer activity, and so on (7). PAB not only induces cancer cell apoptosis, but also inhibits proliferation by blocking the growth of cancer cells at G2/M phase, causing cellular senescence and autophagy, which is associated with the anti-microtubule activity of PAB. PAB modulates the Bcl-2/Bax ratio through reactive oxygen species accumulation and induces caspase-dependent apoptosis via both death receptor and mitochondrial pathways (7). It has been demonstrated that PAB inhibits the growth of *E. multilocularis* by downregulating the TGF-β1 signaling pathway, highlighting its potential as a therapeutic agent for anti-AE treatment (8). Recent pharmacological studies have shown that PAB can regulate the balance of differentiated CD4^+^ T cells (Th1, Th17 and Tregs), which in turn influence the corresponding cytokines to exert immunomodulatory properties (9). Overall, PAB represents a promising novel therapeutic candidate for cancer treatment, immunomodulation, inflammatory disorders, and immunosuppression.

However, a comprehensive understanding of PAB’s effects on the host immune response during infection remains to be fully elucidated. Therefore, this study aims to investigate the *in vitro* effects of PAB on *E. multilocularis* metacestode vesicles, protoscoleces and germinal cells. Furthermore, we will explore the *in vivo* effects of PAB on the functional regulation of T lymphocytes and collagen fibrogenesis in the host tissue surrounding the metacestodes, providing a more complete picture of its potential as a novel therapeutic agent for AE.

## 2 Materials and methods

### 2.1 Ethics and biosafety statements

Animals intraperitoneal injection, parasite infection and euthanasia (cervical dislocation) were carried out in accordance with the Code of Practice for the Breeding and Use of Laboratory Animals issued by the Ministry of Science and Technology of the People’s Republic of China. The study was carried out in compliance with the ARRIVE guidelines. All experimental operations were conducted in accordance with the General Requirements for Laboratory Biosafety of the People’s Republic of China (GB19489-2008). *E. multilocularis* materials were handled in a BSL-3 level laboratory. All experimental procedures were conducted using a Class II biosafety cabinet (ESCO Lifesciences, Changi, Singapore) to maintain a sterile environment. The cabinet was calibrated monthly by a certified technician in accordance with NSF/ANSI 49-2022 standards to ensure optimal airflow and containment. Material containing *E. multilocularis* was autoclaved after sealing and then professionally incinerated.

This study was approved by the Ethics Committee of the Qinghai University Affiliated Hospital (NO. P-SL-2023170). In animal studies, the combination of 50 mg/kg Zoletil 50 and 13.75 mg/kg cyrazine provided anesthetic and analgesic effects. Animals reach the humanitarian endpoint when they show respiratory distress, inability to feed, and inability to stand, and are euthanised by cervical dislocation.

### 2.2 Experimental animals

C57BL/6 mice (female, 18–20 g) were purchased from the Beijing Vital River Laboratory Animal Technology Company (No. 110322220100347742). Mongolian gerbils were purchased from the Hangzhou Medical College Laboratory Animal Center. Gerbils and C57BL/6 mice as susceptible intermediate hosts commonly used in the construction of the secondary *E. multilocularis* animal model.

Animals were housed in standard laboratory conditions (temperature: 22 ± 2°C; relative humidity: 55 ± 5%; 12 h light/dark cycle) in ventilated cages (*n* = 5 per cage). Animals were allowed free access to standard sterile chow and water ad libitum. Sterile dust-free wood chips are used as bedding and replaced every 3 days.

### 2.3 Cell culture and reagents

Human foreskin fibroblasts (HFFs, Cat. No. CL-0352, Pricella biotechnology, Wuhan, China) and Reuber rat hepatocellular carcinoma cells (RH, Cat. No. CL-0195, Pricella biotechnology) were grown in Dulbecco’s Modified Eagle’s Medium (DMEM, Pricella biotechnology) with 10% fetal bovine serum (FBS, Pricella biotechnology) and 1% penicillin–streptomycin (PS, Pricella biotechnology). Normal Human Hepatocyte cells and HK-2 cells were grown in minimum essential medium (MEM, Pricella biotechnology) with 10% FBS and 1% PS (Pricella biotechnology). HFFs, normal human hepatocytes, and HK-2 cells were used to evaluate the toxicity of the drug *in vitro*. RH cells were used for co-culture with metacestode vesicles. All cell lines were maintained at 37°C and 5% CO_2_ in a humidified atmosphere. When the conjunctional level of the cells reached 80–90%, cell cultures were maintained by passaging at a 1:3 split ratio every 2–3 days using 0.25% trypsin–EDTA (Pricella biotechnology) for dissociation. Experiments were performed using cells between passages 3 and 6 to ensure consistent phenotypic characteristics. Pseudolaric Acid B (PAB), albendazole (ABZ), and albendazole sulfoxide (ABZSO) were purchased from Yuanye Bio-Technology (Shanghai, China). The drug PAB was dissolved in PBS (0.01 M PBS, pH = 7.2) containing 10% dimethyl sulfoxide (DMSO, Solarbio, Beijing, China) to a stock concentration of 100 mM. ABZSO was dissolved in DMSO to prepare a 5 mg/mL stock solution. The final concentration of DMSO in all experiments was kept at ≤0.1%. The stock solution was stored at-80°C in the dark to prevent degradation.

### 2.4 Isolation of *Echinococcus multilocularis* protoscoleces

Larval material (isolate Qinghai) was originally isolated from a naturally infected plateau pika (*Ochotona curzoniae*) collected in Yushu, Qinghai Province, China. *E. multilocularis* metacestodes were prepared and cultured as described previously (10). Briefly, Gerbils were anaesthetised and euthanised by cervical dislocation, and later placed in 75% ethanol for 5 min for disinfection. *E. multilocularis* metacestode tissue was removed from the gerbils’ abdominal cavities in a biosafety cabinet (ESCO Lifesciences). The lesions were placed in normal saline (NS) and sliced, and they were then filtered through four layers of gauze into a 50 mL sterile centrifuge tube. Separated protoscoleces were washed 8–10 times with NS at 10 times the volume of the peotoscoleces. Subsequently, the suspension of protoscoleces was filtered with a 40 μm cell strainer to remove the calcified bodies, followed by the collection the naturally settled protoscoleces. Protoscoleces were then collected and activated by incubation in 0.05% w/v pepsin (pH = 2.0 in DMEM) for 30 min at 37°C and 125 rpm according to Fernández et al. as follows (11). The parasite vitality was evaluated by 0.1% methylene blue. Only protoscolex batches exhibiting >98% viability were used. Sterility was confirmed by incubating the protoscolex supernatant on blood agar plates for 48 h to check for bacterial contamination. These protoscoleces were used to inoculate the C57BL/6 mice intraperitoneally and *in vitro* culture. All procedures were performed in a biosafety cabinet using sterile equipment and solutions.

### 2.5 *In vitro* cultivation of *Echinococcus multilocularis* metacestode vesicles

Metacestode vesicles of the isolate Qinghai were cultured as described by Brehm (10, 12), which has been widely validated for maintaining metacestode integrity and growth. The *E. multilocularis* metacestodes were resected from female C57BL/6 mice after 2 months of infection for *in vitro* cultivation. The mice were euthanized via cervical dislocation and sterilized via immersion in 75% ethanol for 5 min; then, they were dissected in a biosafety cabinet (ESCO Lifesciences) and the metacestode tissue material was removed from the abdominal cavity, clipped, and pressurized through a metal tea strainer and incubated in phosphate-buffered saline (0.01 M PBS, pH = 7.2) containing 1% penicillin–streptomycin overnight. Then, 1 mL metacestode tissue was co-cultured with 5 × 10^6^ RH cells in DMEM containing 10% FBS and 1% PS at 37°C in a 5% CO_2_ incubator (HERAcell 150i, ThermoFisher Scientific, Waltham, MA, United States) after five washes with ten times the volume of NS, with medium changes twice a week. The stained vesicles and collapsed vesicles were eliminated by phenol red in DMEM. *In vitro*-cultured metacestode vesicles were used for the experiments when the diameters of the metacestodes reached 2–4 mm, and they were then incubated in pure nitrogen for one week. *E. multilocularis* metacestode vesicles previously cultured *in vitro* were used to isolate the germinal cells through digestion with 0.125% trypsin solution (Pricella Biotechnology). Some of the metacestode vesicles were fixed with 4% paraformaldehyde for pathologic identification.

### 2.6 Confirmation of the protoscoleces and metacestode vesicles via reverse transcription–polymerase chain reaction

In order to determine whether isolated protoscoleces and cultured metacestode vesicles were *E. multilocularis* species, molecular identification was performed according to the method described in Li et al. (13). The echinococcus Cox1 gene, Echinococcus granulosus nad1 gene, and *E. multilocularis* nad5 gene were used to identify the species of protoscoleces and metacestode vesicles. The use of RT-PCR was chosen to confirm the absence of host cell contamination due to its high sensitivity and specificity in detecting nucleic acids. Briefly, the DNA of the protoscoleces, metacestode vesicles and germinal cells was extracted using the TIANamp Genomic DNA Kit (TianGen Biotech, Beijing, China) according to the manufacturer’s instructions. The specific primers were synthesized by the Shanghai Sangong Biological Company and the primer sequences were Nad5 (forward: 5′-CATTAATTATGGATGTTTCC-3′; reverse:5′-GGAAATACCCCACTATCC-3′); Nad1 (forward: 5′-GGTTTTTTATCGGTATGTTGGTGTTAGTG-3′; reverse: 5′-CATTTCTTGAAGTTAACAGCATCACG-3′); cox1 (forward: 5′-TTGAATTTGCCACGTTTGAATGC-3′, reverse: 5′-GAACCTAACGACATAACATAATGA-3′). The RT-PCR was performed on a calibrated and regularly maintained Mastercycler Nexus PCR apparatus (Eppendorf, Hamburg, Germany) according to manufacturer’s specifications. The RT-PCR reaction was performed in a total volume of 25 μL: 10 μL of 2 × Taq PCR mix, 0.6 μL of each primer (10 μM), 2 μL of the DNA product, and 6.8 μL of double-distilled water. Amplification was performed under the following conditions: 94°C for 3 min, 94°C for 30 s, 61°C for 35 s, 72°C for 30 s for 30 cycles, and 72°C for 5 min as the final extension step. The amplified PCR products were separated via 2% agarose gel electrophoresis and photographed.

### 2.7 Confirmation of metacestode vesicles and germinal cells without host cell contamination

The host cell contamination was determined through the amplification of the GAPDH gene of *E. multilocularis* and mouse (14). Total RNA was extracted from the metacetodes and germinal cells according to the instructions of the manufacturer of the RNA Simple Total RNA Kit (TianGen Biotech). RNA was reverse-transcribed to cDNA using the FastKing gDNA Dispelling RT SuperMix (TianGen Biotech) according to the instructions. Reaction solution (25 μL): 10 μL 2 × Taq PCR Mix, 0.5 μL of each primer (10 μM), 2 μL of cDNA solution, 7 μL of double-distilled water. Specific primers were synthesized by the Shanghai Sangong Biological Company, and the primer sequences were Em GAPDH, forward: 5′-CTGCTGACGCTCCCATGTTCGTC-3′; reverse: 5′-CGCCACGCCTTCTTCGAGGG-3′; Mouse GAPDH, forward: 5′-CGTGGGGCAGCCCAGAACAT-3′; reverse: 5′-GAGCAATGCCAGCCCCAGCA-3. The PCR reaction was performed on a Mastercycler Nexus PCR instrument (Eppendorf, Hamburg, Germany) under the following conditions: 94°C for 3 min, 94°C for 30 s, 62°C for 30 s, 72°C for 30 s for 30 cycles, and 72°C for 5 min. The amplified PCR products were separated via electrophoresis on 2% agarose gel and stained with GeneRed (TianGen Biotech) for visualization under ultraviolet light.

### 2.8 PAB toxicity in mammalian cells

The CCK-8 assay (Elabscience, Wuhan, China) was used to assess the toxicity of PAB in mammalian cells *in vitro*. Briefly, HFFs, HK2 cells, or normal human hepatocytes in the logarithmic growth phase were digested and adjusted to 5 × 10^5^ cells/mL. Then, 100 μL of the cell suspension was inoculated into 96-well cell culture plates and incubated at 37°C for 8 h in a 5% CO_2_ incubator. The cells adhered to the wall and different final concentrations of PAB (2.5, 5, 10, 20, 40, 80, 160, 320 μM) were added to interfere with the cells for 48 h. Subsequently,100 μL of new medium and 10 μL of CCK8 solution were added to each well and they were incubated for 1 h. Cell viability was determined by CCK8 assay and expressed as OD450 values (blank subtracted). Cells treated with 0.1% DMSO served as the untreated control (0 μM) to represent the baseline condition. Median inhibitory concentration (IC50) values were calculated using the online IC50 Calculator tool^1^ after logit-log transformation, and averages and standard deviations of six independent replicates were calculated.

### 2.9 PAB against *Echinococcus multilocularis* metacestode vesicle

Phosphoglucose isomerase (PGI) assays were performed as previously described (15). Briefly, Metacestode vesicles with diameters ranging from 2 to 4 mm were isolated through a purification process involving a 2% sucrose solution, followed by multiple rinsing stages using PBS (0.01 M, pH = 7.2). Purified metacestode vesicles were added with phenol red-free DMEM and dispensed into 48-well cell culture plates. Different concentrations of PAB (0.5, 1.0, 2.5, 5, 10, 20, 40, 80, 120, 160, 320 μM) were added and the plates were incubated for 5 days. PGI with 100% release was used as a positive control group in 0.1% Triton X-100 in phenol red-free DMEM. Vesicles treated with 0.1% DMSO served as negative control (0 μM group). The medium supernatant was collected after 5 days of incubation and stored at-80°C for PGI content determination. A PGI assay kit (Abcam, Cambridge, United Kingdom) was used to test the PGI content according to the manufacturer’s instructions. The detection sensitivity of the kit was 0.04 mU/well. The absorbance value at 340 nm was read using an Infinite M200 reader (TECAN, Männedorf, Zürich, Switzerland). The rate of PGI release (PGI%) = (OD value of PAB group-OD value of blank wells)/OD value of Triton X-100 group×100%. Compounds were considered active when they reached 20% of PGI release relative to Triton X-100. The median effect concentration (EC50) values were calculated using an online EC50 calculator^2^ after logit-log transformation, and averages and standard deviations of three independent replicates were calculated. Some vesicles were fixed with 2.5% glutaraldehyde solution and used for SEM and TEM observation.

### 2.10 PAB against *Echinococcus multilocularis* protoscoleces

The protoscoleces were activated with 0.05% w/v pepsin (pH 2 in DMEM) for 30 min at 37°C (11). The activated protoscoleces were obviously valgus. Activated protoscoleces were resuspended in serum-free RPMI1640 medium, and the concentration of protoscoleces was adjusted to 1,000 /mL. A 3 mL suspension was pipetted into a 6-well cell culture plate and 3 mL PAB-containing RPMI1640 medium was added to achieve a final drug concentration of 2.5, 5, 10, 20, 40, 80, 160, or 320 μΜ. As negative control (0 μΜ group), 0.1% DMSO was added accordingly. Moreover, 5 μg/mL albendazole sulfoxide (ABZSO) was used as a positive control (16). The protoscoleces were cultured continuously for 7 days in an incubator at 37°C and 5% CO_2_. A 0.5 mL suspension was aspirated into an EP tube every day and the supernatant was discarded after the protoscoleces had naturally precipitated. 10 μL of pooled protoscoleces was transferred over a slide and mixed by 10 μL of 0.1% eosin and was evaluated after 3 min. Dead protoscoleces absorbed eosin and colored red but alive protoscoleces remained colorless. Subsequently, the protoscoleces were observed under a BX53 inverted microscope (Olympus, Tokyo, Japan). The number of surviving protoscoleces among 100 protoscoleces was counted. The protoscoleces were fixed in 2.5% glutaraldehyde solution and used for further SEM and TEM observation. The experiments were repeated independently three times.

### 2.11 Assessment of PAB effects on *Echinococcus multilocularis* germinal cells

*E. multilocularis* germinal cells were obtained from *in vitro*-grown metacestode vesicles based on the protocol described by Brehm K (17). Briefly, 20 units of cells were distributed among black 384-well plates and different concentrations of PAB (0.5, 1.0, 2.5, 5, 10, 20, 40, 80, 100, 160, 320 μM) were added. The 0.1% DMSO group (0 μM group) was used as the untreated group. After culture at 37°C for 5 days under humidified nitrogen, 25 μL of CellTiter-Glo containing 1% Triton X-100 was added. The cells were incubated at room temperature in the dark for 15 min. After the cells were completely disrupted, the luminescence was read using an Infinite F200 reader (Tecan). CellTiter-Glo results are presented as a percentage of the 0 μM group, representing relative cell viability. IC50 values were calculated using the online IC50 Calculator tool (see text footnote 1) after logit-log transformation, and averages and standard deviations of four biological replicates.

### 2.12 Labeling and detection of 5-ethynyl-2′-deoxyuridine

Short-term labeling and the subsequent whole-mount detection of the thymidine analogue 5-ethynyl-2′-deoxyuridine (EdU, Beyotime Biotechnology, Shanghai, China) were performed as described previously (18). Briefly, germinal cells were seeded in 96-well cell culture plates with about 500 cells per well (100 μL). Germinal cells were added with a final concentration of 10 μM PAB to intervene with 3 days. Germinal cells without drug were used as untreated group (0 μM PAB). After 3 days of intervention, a final concentration of 50 μM EdU was added and incubation was continued for 5 h at 37°C in a 5% CO_2_ incubator. Subsequently, cells were collected by centrifugation at 1000 g and fixed at room temperature for 1 h by adding 4% paraformaldehyde. After the cells were washed with PBS (0.01 M, pH = 7.2), the subsequent staining steps were carried out according to the instructions of the Click-iT^®^ EdU Alexa Fluor^®^ 555 Imaging Kit (Beyotime Biotechnology). After staining, the cells were transferred to a slide and observed under confocal microscopy (ZEISS LSM880, Oberkochen, Germany). A confocal microscope (ZEISS LSM880, Oberkochen, Germany) was used to randomly photograph five fields. Background fluorescence was corrected by subtracting the mean fluorescence intensity of EdU-negative controls (cells without EdU treatment). The ratio of EdU^+^ cells was defined as the number of EdU^+^ nuclei divided by the total number of nuclei using Image J 1.51j8 software (National Institutes of Health, Bethesda, MD, United States). Experiments were performed with three biological replicates.

### 2.13 Glucose consumption

Glucose consumption in *E. multilocularis* protoscoleces and germinal cells was detected using the Screen Quest Colorimetric Glucose Uptake Assay Kit (AAT Bioquest, Sunnyvale, CA, United States). Glucose consumption experiments were performed as previously described (19). Briefly, 200 protoscoleces were inoculated on 48-well cell culture plates or 5,000 germinal cells were inoculated on 96-well cell culture plates. Protoscoleces or germinal cells were treated with 10 μM PAB for 48 h. The protoscoleces or germinal cells without PAB intervention were used as control group (0 μM PAB). Albendazole sulfoxide (ABZSO, 5 μg/mL) was used as a positive control (16). Results are presented as a percentage relative to the values obtained without treatment. Furthermore, subsequent experimental steps were carried out according to the instructions of Screen Quest Colorimetric Glucose Uptake Assay Kit. Finally, monitor the absorbance ratio increase at 570 nm with an absorbance plate reader. Relative glucose consumption was calculated using the following formula: glucose consumption (%) = [(Glucose_0μM_-Glucose_PAB/ABZSO_)/Glucose_0μM_] × 100%. Experiments were performed with three biological replicates.

### 2.14 Evaluation of PAB toxicity *in vivo*

Sixteen 6-8-week-old female C57BL/6 mice were randomly divided into two groups to evaluate the hepatotoxicity and nephrotoxicity of PAB (6 mice/group: Control group, 0.3 mL saline gavage was given daily); PAB group, 0.3 mL PAB (40 mg/kg) gavage was given daily. Mouse blood was collected under anesthesia via the eyeballs and hemocyte analysis were measured using an automated veterinary blood cell analyzer (BC-5000 Vet, Mindray, Shenzhen, China). A portion of blood samples were collected and allowed to clot at room temperature for 1 h before centrifugation at 1500 g for 15 min at 4°C to obtain serum. Serum alanine aminotransferase, aspartate aminotransferase, total bilirubin, direct bilirubin, indirect bilirubin, total protein, albumin, alkaline phosphatase, creatinine, and blood urea nitrogenand were measured by automatic biochemical analyzer (Chemray 800, Rayto, Shenzhen, China). Calibration and quality control procedures were performed according to the manufacturer’s guidelines to ensure data accuracy and reliability. The liver and kidneys were resected from the mice after cervical dislocation and fixed in 4% paraformaldehyde solution for 3 days. The tissues were prepared into 4 μm slices after dehydration and embedding. The pathological changes of liver and kidneys were observed after HE staining.

### 2.15 Evaluation of PAB against *Echinococcus multilocularis in vivo*

C57BL/6 mice were infected intraperitoneally with 200 μL of clipped and resuspended metacestode vesicles, while another 8 mice were injected with 200 μL of normal saline as a control group. Drug administration was performed once a day at a fixed time point. The infected mice were randomly divided into four groups after 4 weeks (8 mice/group): Untreated group, 0.3 mL normal saline gavage treatment was given daily; albendazole (ABZ) group, 0.3 mL 100 mg/kg albendazole gavage treatment was given daily (20); PAB 10 group, 0.3 mL 10 mg/kg PAB gavage treatment was given daily (8); PAB 40 group, 0.3 mL 40 mg/kg PAB gavage treatment was given daily (8). After 4 weeks of drug treatment, blood samples were collected from the eyeballs before euthanasia with cervical dislocation. Subsequently, the mice were immersed in 75% ethanol for 5 min. The parasitic tissue and spleen from each mouse was completely resected and weighed in the biosafety cabinet. The mass of the resected parasitic tissue was used for statistical analyses of the experiment to evaluate the treatment effect. Changes in splenic index reflected the strength of the immune response and the state of the immune function (21). Spleen index = [spleen weight (mg)/(mouse body weight-metacestodes weight) g]. Host tissues surrounding the metacestodes were fixed with 2.5% glutaraldehyde and subsequently examined by TEM for collagen deposition and ultrastructural changes in organelles (specific specimen preparation as described later) The separated tissue was stored at-80°C for subsequent experimental studies.

### 2.16 Flow cytometry

Single-cell spleen suspensions were prepared and analyzed for lymphocyte subsets, Th1, Th2, Th17, and Treg cells (22). Briefly, the spleen was cut up and placed on a 70 μm cell strainer and the spleen was gently milled with a 5 mL syringe plunger. A small amount of pre-cooled PBS (0.01 M, pH = 7.2, Solarbio) was added while grinding until the spleen was completely transformed into a cell suspension, which was collected into a 15 mL centrifuge tube. For metacestode tissues, the tissues were cut into approximately 1 mm^3^ tissue blocks using scissors. Digestive solution containing collagenase II (0.5 mg/mL, Solarbio) and DNasel (0.1 mg/mL, Solarbio) was prepared using RPMI1640 medium (Procell), and 1 cm^3^ tissue blocks were added to 10 mL of digestive solution and digested by shaking at 37°C for 60 min. At the end of the digestion, the cell suspension was filtered through a 200 mesh screen, and centrifuged at 1500 g at room temperature for 5 min, and the cellular precipitate was resuspended with 5 mL of PBS (0.01 M, pH = 7.2, Solarbio). Subsequently, 5 mL of Ficoll (1.077 ± 0.001 g/mL, Solarbio) was added to a new centrifuge tube, and 3 mL of spleen/tissue single-cell suspension was carefully added to the level of the Ficoll separately. The cells were centrifuged in a horizontal centrifuge (5810R, Eppendorf) at 800 g for 30 min at room temperature, with both acceleration and deceleration rates set to 0. At the end of the centrifugation, the milky-white lymphocyte layer was aspirated into a new 15 mL centrifuge tube, mixed with 10 mL of PBS (0.01 M, pH = 7.2, Solarbio) and then centrifuged at 300 g for 10 min. The supernatant was discarded and the cells were washed twice with 10 mL of PBS (0.01 M, pH = 7.2, Solarbio) in a repetitive manner.

For the lymphocyte subsets analysis, 1 × 10^6^ cells were placed in flow tubes and centrifuged at 1000 g for 5 min at room temperature. Then, cells were resuspended in flow cytometry staining buffer (PBS containing 2% FBS) and stained with CD3ε-V450 (17A2, MULTI SCIENCES, Hangzhou, China, 5 μL), CD4-APC (GK1.5, MULTI SCIENCES, 5 μL), and CD8a-PE (2.43, MULTI SCIENCES, 5 μL) in a final volume of 100 μL. The cells were gently vortexed and incubated for 30 min at 4°Cin the dark. Cells were washed with 1–2 mL of flow cytometry staining buffer and centrifuged at 300 g for 5 min. After discarding the supernatant, cells were resuspended in 300 μL of flow cytometry staining buffer. The samples were detected using a NovoCyte flow cytometer (ACEA Viosciences, CA, United States). Flow cytometry data were analyzed using NovoExpress 1.6.2 Software (ACEA Viosciences).

Lymphocyte suspensions were inoculated at a density of 1 × 10^6^ cells/mL in 24-well plates containing 10% FBS + RPMI1640 complete medium and stimulated with PMA/lonomycin (250×, MULTI SCIENCES) and BFA/Monensin (250×, MULTI SCIENCES) for 6 h at 37°C. Following stimulation, cells were harvested and washed with 2 mL flow cytometry staining buffer. After centrifugation at 300 g for 5 min, the supernatant was discarded, and the cells were resuspended in 100 μL of staining buffer.

For surface staining, a total of 5 μL each of CD3e-redFluor 710 (17A2, MULTI SCIENCES) and CD4-APC-Cy7 (GK1.5, MULTI SCIENCES) antibody was added, and the cells were incubated for 30 min at 4°C in the dark. After surface staining, cells were fixed and permeabilized using a fixation/permeabilization kit (MULTI SCIENCES) according to the manufacturer’s instructions. Intracellular cytokine staining was performed using 5 μL IFN-*γ*-PE (XMG1.2, MULTI SCIENCES), 5 μL IL-4-APC (11B11, MULTI SCIENCES), and 5 μL IL-17A -BL421 (4B10, Biolegend, San Diego, CA, United States) for 30 min at 4°C in the dark. After incubation, cells were washed twice with flow cytometry staining buffer by centrifugation at 300 g for 5 min, and the supernatant was carefully discarded. The cells were then resuspended in 300 μL of staining buffer for acquisition. Flow cytometric data were acquired using a BD FACSCelesta flow cytometer (BD Biosciences, Franklin Lakes, NJ, United States). Flow cytometry data were analyzed using FlowJo 10.8.1 software (BD Biosciences).

For Treg cell analysis, 100 μL of cell suspension was added to a flow cytometer tube, 5 μL of Anti-Mouse CD4-APC-Cy7 (GK1.5, MULTI SCIENCES) and 5 μL of CD25-APC (PC61.5, MULTI SCIENCES) or 5 μL of nouse IgG1-APC-Cy7 and 5 μL of mouse IgG1-APC were added and mixed, then incubated for 30 min at 4°C away from light. Each tube was washed twice by adding 2 mL of flow cytometry staining buffer under 300 g centrifugation for 5 min. The supernatant was discarded and the cells were mixed with 1 mL of Fixation/Permeabilisation Buffer (MULTI SCIENCES) and incubated for 45 min at room temperature away from light. Next, 2 mL of 1 × permeabilization Buffer was added to the cells and centrifuged at 300 g for 5 min at room temperature, and the supernatant was discarded. The precipitate was resuspended with 100 μL of 1× permeabilisation Buffer. The cells were mixed with 5 μL of Foxp3- PE or 5 μL of Mouse IgG1-PE and incubated for 30 min at room temperature away from light. Subsequently, cells were washed twice by adding 2 mL 1× Permeabilization Buffer and centrifuged at 300 g for 5 min at room temperature, and the supernatant was discarded. Finally, the cell precipitate was resuspended with 300 μL of flow cytometry staining buffer. Flow cytometric data were acquired using a BD FACSCelesta flow cytometer (BD Biosciences). Flow cytometry data were analyzed using FlowJo 10.8.1 software (BD Biosciences).

Cells were incubated separately with corresponding isotype control antibodies: PE Rat IgG2b *κ* (RTK4530), APC Rat IgG2b κ (TK4530), PerCP/Cyanine5.5 Rat IgG2b κ (RTK4530), APC/Cyanine7 Rat IgG2b κ (RTK4530), PE Rat IgG1 κ (RTK2071), APC Rat IgG1 κ (RTK2071), Brilliant Violet 421™ Rat IgG1 κ (RTK2071), APC Rat IgG1 *λ* (G0114F7), and PE Mouse (IgG1 κ) at the same concentrations as their respective primary antibodies. Isotype-matched antibodies were used as controls to determine background fluorescence and non-specific binding. All isotype control antibodies were purchased from biolegend. Besides, unstained cells served as negative controls, and single-stained samples were used to perform compensation. All flow cytometry analysis was conducted with seven independent biological replicates.

### 2.17 Enzyme-linked immunosorbent assay

The serum and the host tissue surrounding the metacestodes IL-4 (Elabscience, Wuhan, China), IL-10 (Elabscience) and IFN-*γ* (Elabscience) cytokine levels were measured using commercial ELISA kits according to the manufacturer’s instructions. Briefly, blood samples were collected and allowed to clot at room temperature for 1 h before centrifugation at 1500 g for 15 min at 4°C to obtain serum. Tissue samples (100 mg) were homogenized in 0.9 mL ice-cold PBS (0.01 M, pH = 7.2) containing protease inhibitors using a mechanical homogenizer (TianGen Biotech). The homogenates were centrifuged at 12,000 g for 20 min at 4°C, and the supernatants were aliquoted and stored at-80°C until analysis. All reagents and samples were brought to room temperature according to the manufacturer’s instructions. The standards were serially diluted with the provided diluent to prepare the standard curve. Meanwhile, the samples were diluted according to the results of the pre-tests to ensure that their concentrations were within the standard curve. Add 100 μL of standard, sample or blank (sample diluent) to each well of the plate and incubate at 37°C for 90 min. Discard the liquid in the wells, add 100 μL of biotinylated detection antibody working solution to each well, and incubate at 37°C for 1 h. After discarding the liquid in the wells, each well was rewashed three times by adding 350 μL of washing solution. Following washing, the HRP-conjugate working solution was added 100 μL per well and incubated at 37°C for 30 min. After the final wash, TMB substrate was added and the reaction was stopped with a termination solution. The optical density (OD) was measured at 450 nm using a microplate reader (Infinite M200 reader, TECAN, Männedorf, Switzerland). The standard curve was plotted and analyzed using OriginPro 8.5.0 software (OriginLab Corporation, Northampton, MA, United States). The standard curve was valid for use as the absorbance values showed a strong linear relationship (*R*^2^ > 0.99) within the tested concentration range. Sample concentrations were calculated by interpolating the OD values on the standard curve. All samples were analyzed in duplicate, and the background values (blank wells) were subtracted from all readings.

### 2.18 Hematoxylin–eosin staining

The metacestode tissues, livers, and kidneys were fixed in 4% paraformaldehyde for 48 h at room temperature. Dehydration was carried out using increasing concentrations of ethanol (70, 80, 90, 95%, and twice in 100%, each for 1 h at room temperature). Tissues were then cleared in two changes of xylene for 15 min each. Subsequently, samples were infiltrated with paraffin wax at 65°C for 2 h, followed by embedding in fresh paraffin wax. Paraffin sections were cut at 4 μm thickness using a microtome (HistoCore BIOCUT, Leica Biosystems, Wetzlar, Germany). Sections were deparaffinized in xylene (3 × 5 min) and rehydrated through a descending ethanol series (100, 95, 80, 70%, 5 min each) to distilled water. Sections were stained with 0.5% hematoxylin (Servicebio, Wuhan, China) for 5 min, followed by a 10 min rinse in running tap water to remove excess stain. Differentiation was performed using 1% acid alcohol for 3 s. After a quick rinse in tap water, the sections were then blued in 0.2% ammonia water for 30 s. Following another tap water rinse, the sections were counterstained with 0.5% eosin (Servicebio) for 1 min. Subsequently, sections were dehydrated through a graded ethanol series (95% ethanol for 2 min, followed by two changes of 100% ethanol for 2 min each) and cleared in two changes of xylene for 3 min each. Finally, the stained sections were then mounted using a neutral gum and a coverslip. HE-stained sections were observed and imaged using a BX53 microscope (Olympus) with a FL-20 digital camera (XingtuSun company, Shenzhen, China). Images were taken at 200× total magnification.

### 2.19 Masson trichrome staining

Tissue sections were stained to assess collagen deposition and fibrosis according to the instructions of the Masson trichrome stain kit (Solarbio). Briefly, paraffin sections (4 μm) were prepared as described above. Sections were deparaffinized in xylene and rehydrated through graded ethanol series (100, 95, 80, and 70%) to distilled water. Sections were stained using Weigert’s iron hematoxylin staining solution (1% hematoxylin in 95% ethanol) for 10 min and distilled water washed to remove excess staining solution. Next, the sections were differentiated with 1% acid differentiation solution for 10 s, and washed with distilled water for 30 s. Subsequently, the sections were reblued with bluing solution for 3 min, and washed with distilled water for 30 s. Following this, the sections were stained with Ponceau-Acid fuchsin solution for 6 min, followed by dropwise washed of weak acid solution for 30s. The excess liquid was poured off, and the sections were treated with phosphomolybic acid solution for 1 min, followed by washing with weak acid working solution for 30 s. Excess liquid was poured off, and the sections were stained with 2.5% aniline blue solution for 1 min and washed with weak acid solution for 30 s. Finally, the sections were dehydrated by 95% ethanol for 3 s, anhydrous ethanol for two times for 5 s each, xylene clear for two times for 2 min each, and sealed with neutral gum. The stained sections were observed under a BX53 microscope (Olympus) and 200× images were collected. Each sample was randomly photographed in five regions. The extent of collagen deposition was quantified using Image J 1.51j8 software (National Institutes of Health). The collagen volume fraction was calculated as the ratio of collagen-stained area (blue) to the total tissue area, expressed as a percentage.

### 2.20 Periodic acid schiff staining

Sections were stained to identify the laminated layer according to the instructions of the PAS staining kit (Solarbio). Briefly, paraffin sections (4 μm) were prepared as described above. Sections were deparaffinized in xylene and rehydrated through graded ethanol series (100, 95, 80, and 70%) to distilled water. Sections were treated with 0.5% PAS oxidant for 6 min at room temperature, rinsed once in tap water and then soaked twice in distilled water. Subsequently, the sections were stained with Schiff reagent (0.5% basic magenta) for 15 min and rinsed with distilled water for 10 min, then stained with 0.5% hematoxylin solution for 1 min, and differentiated with a drop of acidic differentiation solution for 3 s. The sections were immersed in tap water for 10 min, and finally, the sections were dehydrated step by step in regular ethanol, transparent in xylene, and sealed with neutral gum. Finally, the sections were dehydrated by 95% ethanol for 3 s, anhydrous ethanol for two times for 5 s each, xylene clear for two times for 2 min each, and sealed with neutral gum. The stained sections were observed under a BX53 microscope (Olympus) and 200× images were collected. PAS reaction positive substance showed red or purplish red.

### 2.21 Scanning electron microscopy and transmission electron microscopy analysis

The variations in the microstructure of the protoscoleces or metacestodes were observed under an electron microscope (23). Protoscoleces or metacestode vesicles or host tissues surrounding metacestodes were fixed in 5 mL of 2.5% glutaraldehyde in 0.01 M PBS (pH = 7.2) at 4°C overnight. After washing three times with 0.01 M PBS for 15 min each, specimens were post-fixed with 1% osmium tetroxide for 2 h at room temperature.

For SEM, the specimens were then dehydrated through a graded ethanol series (30% → 50% → 70% → 80% → 90% → 95% → 100%, 15 min each step). Next, samples were treated with hexamethyldisilazane (Sigma-Aldrich, St. Louis, MO, United States) for 10 min, followed by air-drying overnight in a desiccator. The dried samples were then mounted on aluminum stubs, and sputter-coated with a 10 nm gold–palladium layer (E-1045, HITACHI, Tokyo, Japan). Images were acquired using a JSM-IT700HR scanning electron microscope (JEOL, Tokyo, Japan) at an accelerating voltage of 20 kV. Each experiment was carried out independently in triplicate for each group.

For TEM, after washing, specimens were dehydrated through a graded acetone series (30% → 50% → 70% → 80% → 90% → 95% → 100, 100% concentration replaced 3 times). Subsequently, the specimens were sequentially permeated in a 1:1 mixture of acetone and Epon 812 resin for 2 h, a 1:3 mixture for 4 h, and finally placed in pure Epon 812 resin for overnight permeation. The specimens were then embedded in fresh Epon 812 resin and polymerized at 60°C for 48 h. Ultrathin sections (50 nm) were cut with a diamond knife using an ultramicrotome (Leica UC7) and collected on 200 mesh copper grids. The sections were stained with 2% uranyl acetate (SPI-CHEM, West Chester, PA, United States) for 15 min and lead citrate (SPI-CHEM) for 10 min. The stained sections were examined using a JEM-1400FLASH transmission electron microscope (JEOL) operated at 80 kV. Each experiment was carried out independently in triplicate for each group.

### 2.22 Immunohistochemical analysis

Paraffin-embedded tissue sections (4 μm thick) were deparaffinized in xylene and hydrated through graded ethanol solutions. Sections were deparaffinized in xylene and rehydrated through graded ethanol series. For antigen retrieval, deparaffinized sections were immersed in 10 mM sodium citrate buffer (pH 6.0) and heated in a microwave oven (PC2320W, Midea, Foshan, China) at 700 W for 15 min. Slides were then allowed to cool in the buffer for 30 min at room temperature. Next, sections were incubated with 3% H_2_O_2_ solution (Macklin Biochemical Technology, Shanghai, China) for 10 min at room temperature to eliminate endogenous peroxidase activity. After blocking with normal goat serum for 40 min at room temperature, sections were incubated with primary antibodies against cleaved caspase3 primary antibody (1:300, Cell Signaling Technology, Boston, MA, United States) overnight at 4°C. Meanwhile, negative control with PBS (0.01 M, pH = 7.2) and rabbit IgG isotype control (1:300, Cell Signaling Technology) were included to ensure specificity of immunostaining. Following washing with PBS (3 times×3 min), sections were incubated with HRP-conjugated goat anti-Rabbit IgG (1:300, Sangon Biotech, Shanghai, China) for 1.5 h at room temperature. Slides were placed in PBS (0.01 M, pH = 7.2) on a decolourising shaker and shaken and washed 3 times (3 min each). Subsequently, the sections were colour developed using the DAB color development kit (Solarbio). The color developing time was controlled under the microscope, and the positive color was brown and yellow, and the sections were rinsed with tap water to terminate the color development. Sections were re-stained with hematoxylin for 1 min. Finally, sections were observed at 200× magnification, images were taken using BX53 microscope (Olympus). The expression levels of cleaved caspase3 protein were quantified by measuring the average optical density (AOD, AOD=Integratedoption density/Area) of immunohistochemical staining using Image J 1.51j8 software (National Institutes of Health, Bethesda). All morphological analyses were performed by an investigator who was blinded to the experimental groups.

### 2.23 Real-time fluorescence quantitative PCR

Metacestode tissues were collected and immediately frozen using liquid nitrogen and stored at-80°C. The total RNA was extracted using TRIzol reagent (ThermoFisher Scientific) according to the manufacturer’s instructions, followed by DNase I treatment (1 U/μL, ThermoFisher Scientific) at 37°C for 30 min to eliminate genomic DNA contamination. The quantity and quality were assessed using the NanoDrop2000 (ThermoFisher Scientific). The ratios of absorbance at 260/280 (acceptable value range: 1.8–2.0) and 260/230 (acceptable value range: 2.0–2.2) were determined to confirm that all samples were suitable for reliable reverse transcription–quantitative PCR analysis. The samples that do not meet the conditions are re-extracted. The RNA integrity was determined via 1.5% agarose gel electrophoresis. Then, 2 μg of total RNA was converted into cDNA sequences using the FatKing gDNA Dispelling RT SuperMix Kit (TianGen Biotech). The RT-PCR reactions were performed using the SuperReal PreMix Plus (SYBR Green) Kit (TianGen Biotech) on an ABI QuantStudio5 PCR system (Applied Biosystems, Carlsbad, CA, United States). The specific primers sequences are shown in Table 1. The reaction system consisted of 10 μL SuperReal PreMix Plus (2×), 0.6 μL forward primer (10 μM), 0.6 μL reverse primer (10 μM), 0.4 μL ROX (50×), 2 μL cDNA, and 6.4 μL H_2_O. The PCR cycling reaction conditions were as follows: 95°C for 15 min; 95°C for 15 s, 60°C for 30 s. Melting curve analysis was performed immediately after amplification using the following parameters: 95°C for 15 s, 60°C for 1 min, Gradual temperature increase from 60°C to 95°C (0.3°C/step), 95°C for 15 s. Each sample was analyzed in technical triplicates, and the average Ct value was used for quantification. The relative expression levels were normalized to RPS18 and calculated using the 2^−△△Ct^ method (24).

**Table 1:** Primers used for quantitative polymerase chain reaction.

Gene names	Forward (5′-3′)	Reverse (5′-3′)
Collagen I	GACAGGCGAACAAGGTGACAGAG	CAGGAGAACCAGGAGAACCAGGAG
Collagen III	GGTGTAAAGGGTGAACGTGGTAGTC	TTGCCAGGAGGACCAGGAAGAC
RPS18	AGCTGCGTGAGGACCTGGAG	GGCCAGTGGTCTTGGTGTGC

### 2.24 Western blot analysis

The tissue samples (50 mg) were homogenized in 500 μL ice-cold RIPA buffer (1:10, w/v, 25 mM Tris–HCl pH 7.6, 150 mM NaCl, 1% NP-40, 1% sodium deoxycholate, 0.1% SDS, ThermoFisher Scientific) supplemented with protease and phosphatase inhibitors (1:100, Sangon Biotech). Samples were sonicated on ice using a probe sonicator (H150, OuHor, Shanghai, China) at 20% amplitude, with 3 cycles of 3 s on/9 ss off. The samples were then incubated on ice for 30 min. The supernatant was extracted via centrifugation at 12,000 g for 15 min at 4°C. The supernatant was diluted with 5 × protein sampling buffer and boiled at 95°C for 10 min according to the manufacturer’s instructions, and then the protein samples were aliquoted and stored at-80°C. The protein concentration was determined with a BCA protein quantification kit (ThermoFisher Scientific). After this, 30 μg of the protein sample was separated via SDS-PAGE electrophoresis. Then, the proteins were transferred to a 0.2 μm PVDF membrane (Merck Millipore, Darmstadt, Germany) and blocked in 5% BSA (for phosphorylated proteins) or 5% skimmed powdered milk (for non-phosphorylated proteins) in TBST (10 mM Tris, 150 mM NaCl, 0.05% Tween-20, pH = 7.2) for 1 h at room temperature. The membrane was then incubated with the specific antibody: Bax (1:1000, Proteintech, Wuhan, China), Bcl 2 (1:1000, Proteintech), Cleaved caspase3 (1:1000, Cell Signaling Technology), Collagen I (1:1000, Proteintech), Collagen III (1:1000, Proteintech), MMP1 (1:10500, ABclonal, Wuhan, China), MMP2 (1:1000, ABclonal), MMP3 (1:1000, ABclonal), MMP9 (1:1000, ABclonal), MMP13 (1:1000, ABclonal), p-PI3K (1:800, Cell Signaling Technology), PI3K (1:1000, Proteintech), p-AKT (1:1000, Cell Signaling Technology), Akt (1:1000, Cell Signaling Technology), and *β*-actin (1:2000, ABclonal) overnight at 4°C. After primary antibody incubation, membranes were washed three times (10 min each) with TBST and then incubated with HRP-conjugated goat anti-rabbit IgG (1:5000, Proteintech) or HRP-conjugated goat anti-mouse IgG (1:5000, Proteintech) for 1 h at room temperature. Following three additional 10 min washes with TBST (TBS containing 0.1% Tween-20), protein bands were visualized using enhanced chemiluminescence substrate (ThermoFisher Scientific) and detected using a chemiluminescence imaging system (SageCreation, Beijing, China). The exposure time was optimized for each protein to ensure signals were within the linear range. Western blot bands were quantified by densitometric analysis using ImageJ software (Version 1.51j8, National Institutes of Health). For densitometry analysis, rectangular selections of equal size were drawn around each protein band. Background signal was subtracted using the rolling ball algorithm with a radius of 50 pixels. The integrated density of each band was measured and normalized to the corresponding *β*-actin control. Western blot experiments were performed with seven biological replicates.

### 2.25 Statistical analysis

Statistical analyses were performed using GraphPad Prism software (version 8.2.1, GraphPad Software, San Diego, CA, United States). Data are presented as mean ± standard deviation (SD) with error bars indicating standard deviation for normally distributed variables and median with interquartile range (IQR) for non-normally distributed variables. The normality of data distribution was assessed using the Shapiro–Wilk test, and homogeneity of variance was evaluated using Levene’s test. For normally distributed data with homogeneous variance, independent-samples *t*-test was used for comparisons between two groups, while one-way ANOVA followed by Tukey’s *post hoc* test was applied for multiple group comparisons. When data failed to meet the assumptions of normality or homogeneity of variance, non-parametric Kruskal-Wallis test followed by Dunn’s post hoc test was employed. Multiple comparison corrections were performed using Bonferroni correction control. All experiments were conducted with at least three biological replicates. A threshold of *p* < 0.05 was considered statistically significant for all comparisons.

## 3 Results

### 3.1 Cultivation and confirmation of the *Echinococcus multilocularis* metacestodes and germinal cells

**Figure 1:**
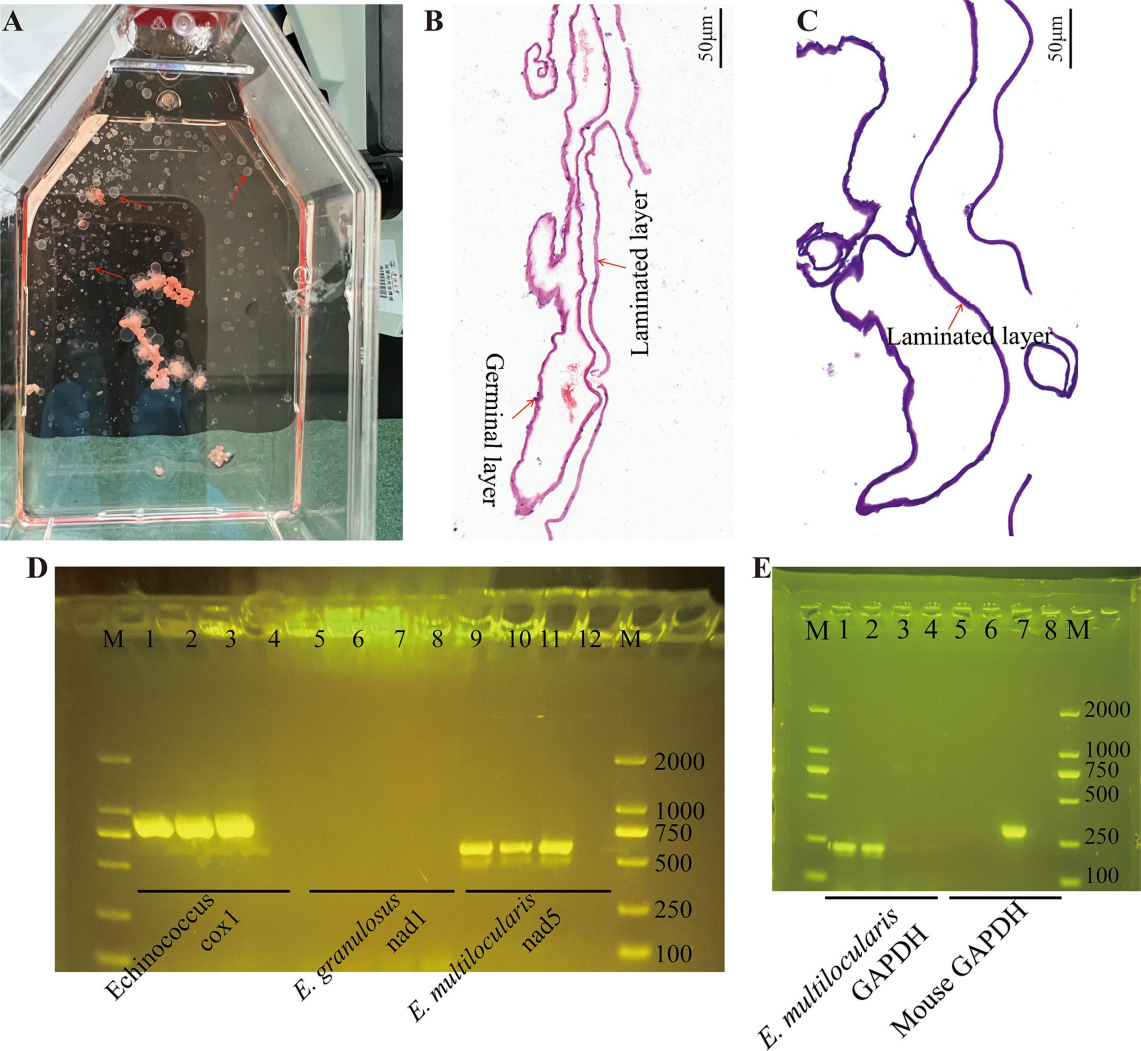
Cultivation and confirmation of the *E. multilocularis* metacestodes and germinal cells. **(A)** Gross morphology of *E. multilocularis* metacestodes cultured *in vitro*. **(B)** The structure of the metacestode vesicles as observed via HE staining. **(C)** The structure of the metacestode vesicles as observed via PAS staining. A distinctly cell-free laminated layer was observed after PAS staining. **(D)** PCR analysis of DNA isolated from protoscoleces, metacestode vesicles, and germinal cell preparations with primers specific to Echinococcus sp. cox1, *E. granulous* nad1, and *E. multilocularis* nad5. Here, 1, 5, and 9 are protoscoleces; 2, 6, and 10 are vesicle tissue; 3, 7, and 11 are germinal cells; and 4, 8, and 12 are negative controls. **(E)** Confirmation of metacestode vesicles and germinal cells with or without host cell contamination. Here, 1 and 5 are vesicle tissue; 2 and 6 are germinal cells; 3 and 7 are mouse liver tissue; and 4 and 8 are negative controls.

Experimental infection of C57BL/6 mice with protoscoleces isolated from gerbils resulted in palpable abdominal lesions after 2 months. Metacestode vesicles were successfully isolated from these lesions and maintained in culture, with visible vesicle formation observed after 2 months. Figure 1A shows the gross morphology of metacestode vesicles cultured *in vitro*. Metacestode vesicles were observed to have a laminated layer with no cellular structure and a germinal layer with cellular structure after HE staining (Figure 1B). The laminated layer structure of the metacestode vesicles was further determined by PAS staining (Figure 1C). Purplish red continuous laminated layer structure observed after PAS staining. Species identification and sample validation were performed through molecular analysis. The gene amplification results confirmed the expression of the *E. multilocularis* cox1 and nad5 genes, while the *E. granulosus* nad1 gene was not expressed in protoscoleces, metacestode vesicles, and germinal cells, thus verifying the specimens as *E. multilocularis* (Figure 1D). The presence or absence of host cell contamination was further clarified by amplification of the *E. multilocularis* and mouse GAPDH genes. The *E. multilocularis* GAPDH gene was amplified in metacestode vesicles and germinal cells, while the mouse GAPDH gene was not amplified (Figure 1E).

### 3.2 *In vitro* activity of PAB against *Echinococcus multilocularis* metacestodes

**Figure 2:**
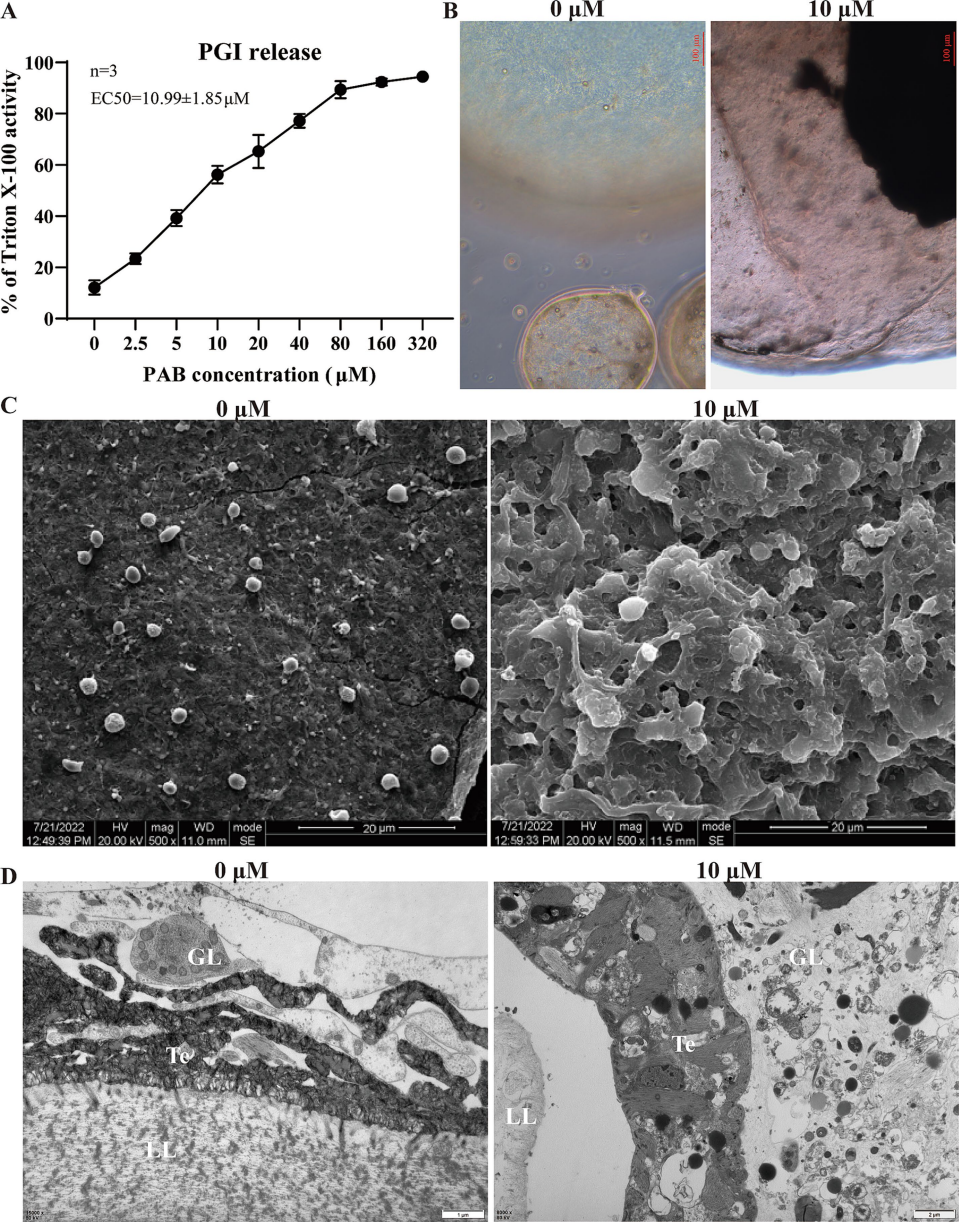
Effect of PAB against *E. multilocularis* metacestodes. **(A)** Determination of PGI content in culture supernatants of metacestode vesicles after PAB intervention via PGI method. The data are presented as the mean ± SD obtained from three independent experiments. **(B)** Morphological observation of metacestode vesicles after PAB intervention. Scale bar = 20 μm. **(C)** SEM observation of metacestode vesicles after PAB intervention. **(D)** TEM observation of metacestode vesicles after PAB intervention. Scale bar = 1 or 2 μm.

The level of PGI release in the culture supernatant was determined after intervention with different concentrations of PAB. of the culture supernatant revealed that PGI levels increased in a concentration-dependent manner with PAB treatment (Figure 2A). The EC50 value of PAB against *E. multilocularis* metacestodes was 10.99 ± 1.85 μM (Figure 2A). Subsequently, morphological observation was performed on the vesicles after 10 μM PAB intervention (Figure 2B), which resulted in the separation of the laminated layer from the germinal layer. Further observations via SEM and TEM showed microstructural changes in the metacestode vesicles after 10 μM PAB intervention. To investigate the effect of PAB intervention on the morphology of the inner surface of the metacestode vesicles, we performed SEM analysis (Figure 2C). SEM micrographs showed that the inner surface of metacestode vesicles in the 0 μM PAB group had many smaller vesicle structures, whereas these characteristic vesicular formations were notably absent in specimens treated with 10 μM PAB, suggesting that PAB caused damage to the inner vesicle structure. The metacestode vesicle structure was further observed by TEM (Figure 2D). The typical structures of the germinal layer, laminated layer, and tegument could be observed in the 0 μM PAB group. The structure of the germinal layer in the 10 μM PAB group was obviously destroyed and apoptotic bodies were observed.

### 3.3 Effect of PAB on the viability of the *Echinococcus multilocularis* germinal cells

**Figure 3:**
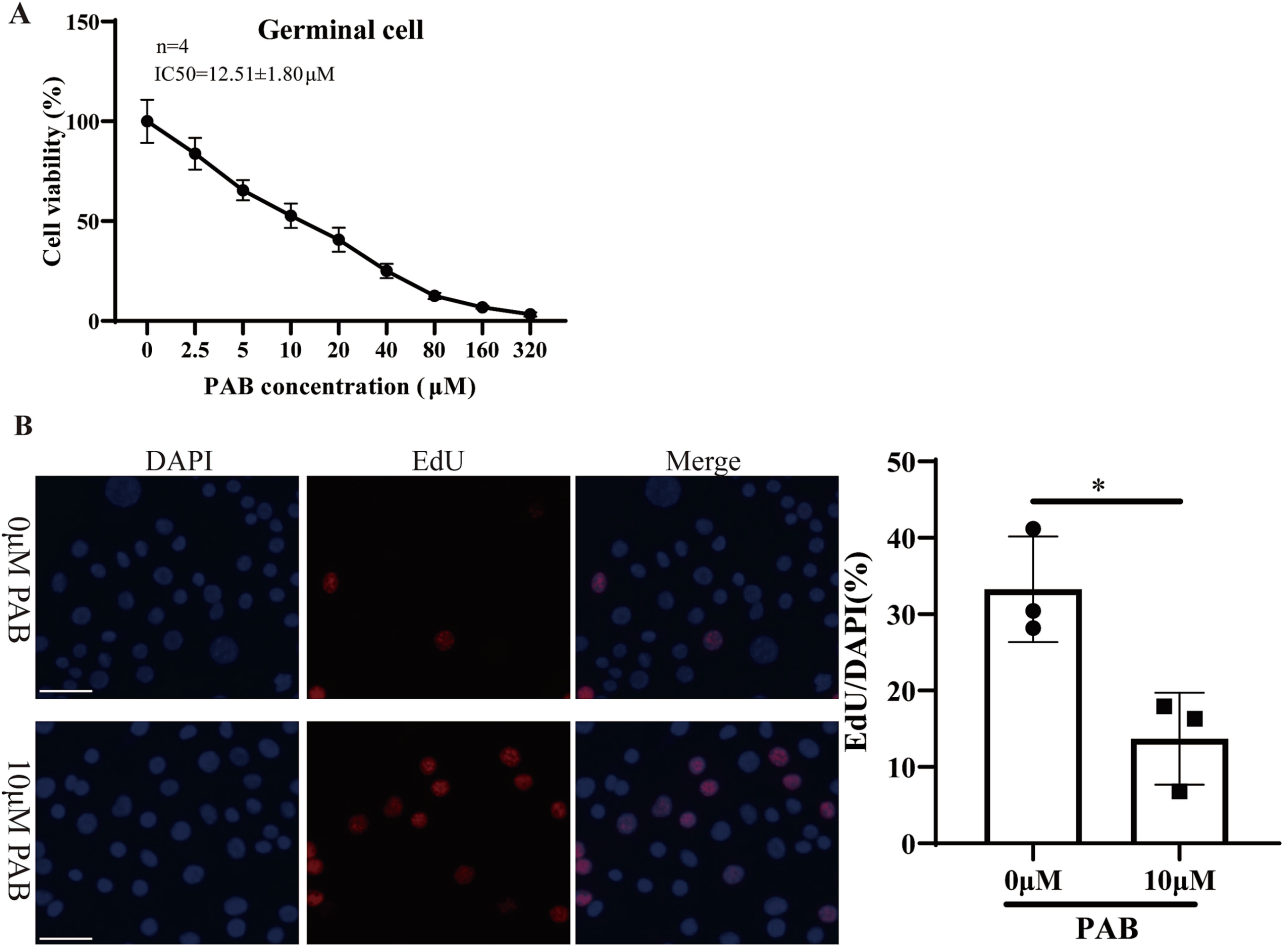
Cytotoxicity of 10 μM PAB on germinal cells. **(A)** The Alamar Blue assay was used to evaluate the viability of the germinal cells after being treated with different concentrations of PAB. The data are presented as the mean ± SD from four independent experiments. **(B)** The EdU method was used to determine the proliferative capacity of the germinal cells. Scale bar = 1 μm.

Within the metacestode vesicles, germinal cells are responsible for the proliferation and differentiation necessary for parasite development. Furthermore, the effect of PAB on the viability of *E. multilocularis* germinal cells was evaluated via the Alamar Blue assay after treatment with different concentrations of PAB (Figure 3A). The results showed that the viability of the germinal cells was gradually reduced with an increase in the PAB concentration. The IC50 value of the effect of PAB on the germinal cells was 12.51 ± 1.80 μM. Subsequently, the proliferative capacity of the germinal cells after 10 μM PAB intervention was detected via EdU labeling (Figure 3B). 10 μm PAB treatment inhibited the proliferation of germinal cells compared to the 0 μm group (*t* = 0.021, *p* < 0.05).

### 3.4 *In vitro* activity of PAB against *Echinococcus multilocularis* protoscoleces

**Figure 4:**
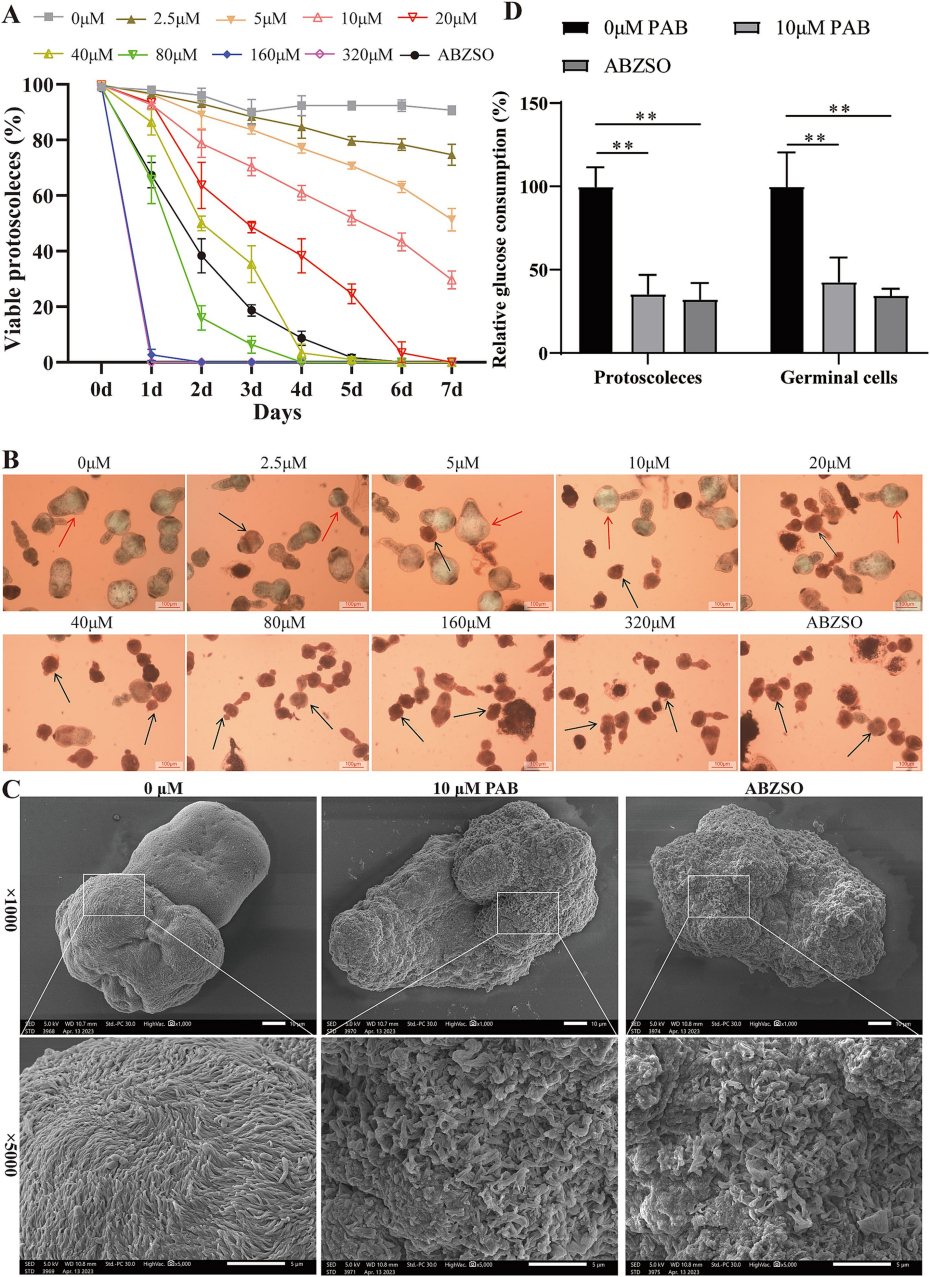
Effects of PAB against *E. multilocularis* protoscoleces. **(A)** The survival rate of protoscoleces after continuous intervention with different concentrations of PAB for 7 days. To evaluate the survival of protoscoleces, a 0.1% eosin staining exclusion method was used daily. The data are presented as the mean ± SD obtained from three independent experiments. **(B)** Gross morphology of protoscoleces after treatment with different concentrations of PAB for 5 days. After treatment with different concentrations of PAB for 5 days, eosin staining was used to observe the protoscoleces. The red arrow shows the live protoscoleces and the black arrow shows the dead protoscoleces. Scale bar = 100 μm. **(C)** SEM observation of protoscoleces treated with 20 μM PAB for 5 days. Scale bar = 10 μm. **(D)** PAB inhibited glucose consumption. The protoscoleces or germinal cells were treated with 10 μM PAB, and the relative glucose utilization was determined. The data are presented as the mean ± SD obtained from three independent experiments. Statistical comparisons were performed using a Tukey’s *post hoc* test. ** Indicates *p* < 0.01.

To examine whether PAB has a direct killing effect on protoscoleces, we tested the effects of different concentrations of PAB on parasite survival. The addition of 2.5–320 μM PAB exhibited concentration-dependent effects on protoscolex survival (Figure 4A). Upon incubation with 2.5–80 μM PAB for 3 days, protoscolex survival rates were 88.33, 83.67, 70.33, 48.67, 35.33, and 6.33%, respectively, with complete lethality observed beyond 160 μM. When incubated with 2.5–10 μM PAB for 7 days, survival rates were 74.67, 51.33, and 29.67%, with total mortality occurring beyond 20 μM. Figure 4B presents representative images of eosin-stained protoscoleces. Next, the damage to protoscoleces as a result of PAB treatment was further demonstrated by SEM (Figure 4C). After 10 μM PAB treatment, the protoscoleces were deformed and the microvilli structure was disorganised. In contrast, there was no significant damage to the structure of the protoscoleces without PAB treatment. Subsequently, we preliminarily assessed whether the anti-*E. multilocularis* effect of PAB was related to glucose consumption (Figure 4D). Compared with 0 μM PAB group, 10 μM PAB and 5 μg/mL ABZSO treatment resulted in reduced glucose consumption in protoscoleces and germinal cells (*p* < 0.01).

### 3.5 Cytotoxicity measurements of PAB on mammalian cells

To assess the toxicity of PAB, the proliferation of human hepatocyte cells, HFFs cells and HK2 cells was first determined *in vitro* using a CCK-8 kit. In this assay, the cells were treated with a concentration series (2.5, 5, 10, 20, 40, 80, 160 and 320 μM) for 48 h (Supplementary Figure S1). The results demonstrated that PAB significantly inhibited the proliferation of normal human hepatocytes (One-way ANOVA: 0.91 ± 0.15 OD at 160 μM and 0.67 ± 0.09 OD at 320 μM, *n* = 6, *p* < 0.01) and HFFs (One-way ANOVA: 0.95 ± 0.13 OD at 160 μM and 0.73 ± 0.08 OD at 320 μM, *n* = 6, *p* < 0.01) compared with the respective 0 μM group. This suggests that at higher concentrations, PAB may significantly impede the growth and proliferation of these cell types. Furthermore, the inhibitory effect of PAB was even more pronounced in HK2 cells, and the cell proliferation rate was significantly reduced in the 80 μM (One-way ANOVA: 0.70 ± 0.06 OD, *n* = 6, *p* < 0.01), 160 μM (One-way ANOVA: 0.46 ± 0.05 OD, *n* = 6, *p* < 0.01) and 320 μM (One-way ANOVA: 0.34 ± 0.06 OD, *n* = 6, *p* < 0.01) groups compared with that in the 0 μM group (0.87 ± 0.13 OD).

### 3.6 *In vivo* toxicity assessment in mice

The *in vivo* toxicity of PAB was evaluated in normal C57BL/6 mice treated with 40 mg/kg PAB for 4 weeks. The WBC, HB and PLT in the whole blood and the levels of ALT, AST, TP, ALB, GLB, T-Bil, D-Bil, I-Bil, ALP, and BUN in the serum are shown in Table 2. A Student’s *t*-test indicated that there were no statistically significant differences in blood cell and biochemical indices between the Control group and the PAB40 group (all *p* > 0.05). Further histological examination using HE staining of liver and kidney tissues from both the PAB40 and Control groups revealed no significant pathological changes. In the liver, both groups displayed normal histological features, including well-preserved hepatocyte architecture, intact hepatic lobules, and absence of inflammatory cell infiltration, necrosis, or fibrosis. Similarly, kidney tissues from both groups showed no signs of damage, with well-maintained glomerular structure, clear renal tubules, and no evidence of tubular degeneration, necrosis, or interstitial inflammation (Supplementary Figure S2).

**Table 2:** Effect of PAB treatment on liver and kidney function in mice (*n* = 6).

Index	Control	PAB 40	*t*	*p-*value
White blood cell (10^9^/L)	7.89 ± 1.11	8.09 ± 0.99	0.3377	0.7425
Hemoglobin (g/L)	128.17 ± 11.65	127.67 ± 15.39	0.0634	0.9507
Platelet (10^9^/L)	485.17 ± 54.81	481.33 ± 74.05	0.1019	0.9208
Total protein (g/L)	59.25 ± 3.05	61.70 ± 4.67	1.0760	0.3700
Albumin (g/L)	25.50 ± 3.27	27.75 ± 3.14	1.2160	0.2520
Globulin (g/L)	34.15 ± 3.09	33.53 ± 2.03	0.4080	0.6919
Alanine aminotransferase (U/L)	61.17 ± 8.06	62.33 ± 6.41	0.2775	0.7870
Aspartate aminotransferase (U/L)	198.67 ± 49.63	194.00 ± 29.37	0.1982	0.8468
Total bilirubin (μmol/L)	0.66 ± 0.18	0.60 ± 0.11	0.5905	0.5680
Direct bilirubin (μmol/L)	0.10 ± 0.04	0.12 ± 0.05	0.7098	0.4941
Indirect bilirubin (μmol/L)	0.54 ± 0.18	0.53 ± 0.15	0.1224	0.9050
Alkaline phosphatase (U/L)	86.50 ± 5.39	85.33 ± 6.80	0.3292	0.7488
Blood urea nitrogen (mmol/L)	9.11 ± 1.19	5.62 ± 2.30	3.2960	0.0081
Creatinine (μmol/L)	16.13 ± 3.20	15.63 ± 1.81	0.3334	0.7457

### 3.7 Effect of PAB against *Echinococcus multilocularis* metacestodes *in vivo*

**Figure 5:**
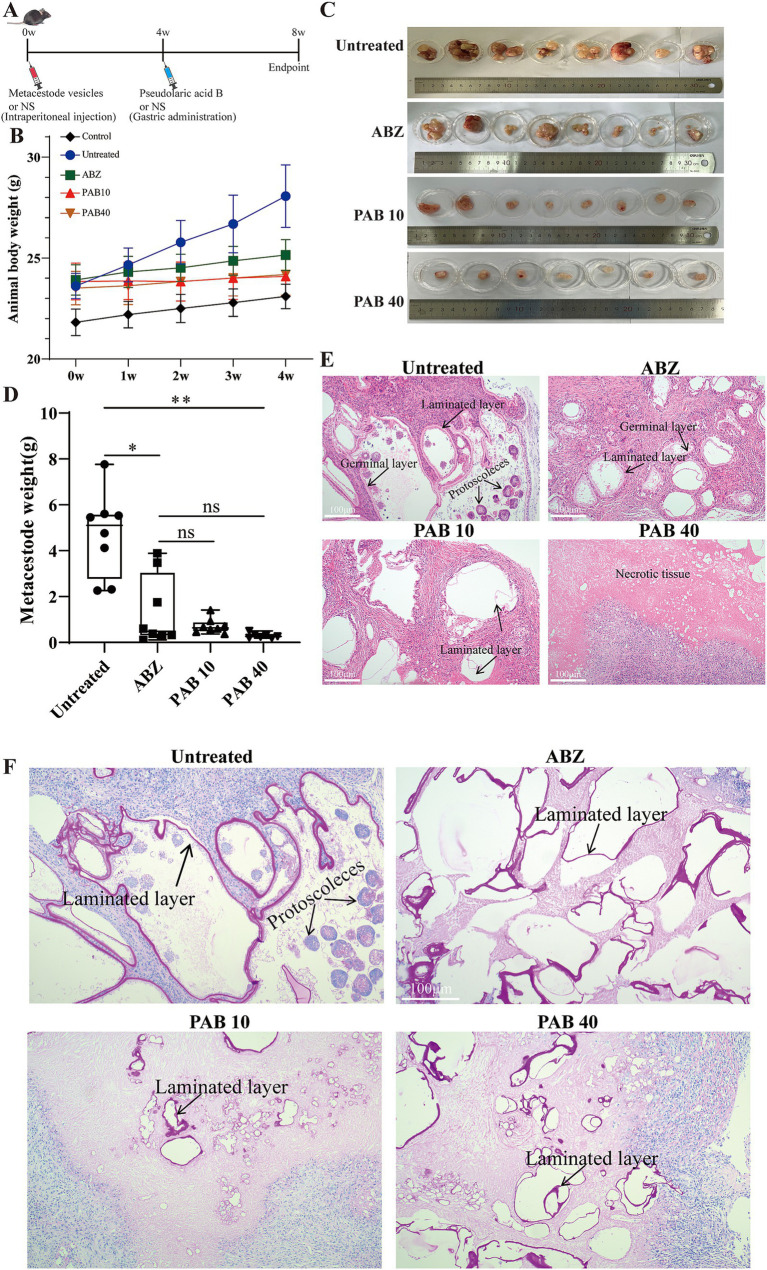
Evaluation the anti-echinococcus effectiveness of PAB *in vivo*. Four weeks after the infection of C57BL/6 mice with *E. multilocularis* metacestode tissue via intraperitoneal injection, the mice received the intragastric administration of PAB for 4 weeks. The positive control was treated with albendazole at 100 mg/kg. **(A)** Schematic diagram of the effect of PAB on mice with secondary AE infection. **(B)** Changes in body weight of mice during PAB treatment. **(C)** Images of metacestodes resected from different groups. **(D)** Parasite wet weight in different groups. The data are presented as median (IQR) from seven or eight independent experiments. Statistical comparisons were performed using a Dunn’s post hoc test. **(E)** HE staining of *E. multilocularis* metacestodes in different groups. Scale bar =100 μm. **(F)** PAS staining of laminated layer in different groups. The sections show the PAS-positive laminated layers of *E. multilocularis* metacestodes. Scale bar = 100 μm. * Indicates *p* < 0.05. ** Indicates *p* < 0.01.

On the basis of PAB toxicity evaluation, the *in vivo* impact of treatment on parasite load was investigated in a C57BL/6 mouse model of *E. multilocularis* protoscoleces infection (Figure 5A). One mouse in the PAB40 group died from asphyxiation due to liquid reflux. No significant increase in body weight was observed in the mice following treatment with ABZ and PAB (Figure 5B). In addition, Figures 5C,D shows the parasite load after PAB treatment. The wet weight data of cysts in the ABZ group was not normally distributed according to Shapiro–Wilk test (W = 0.7734 *p* = 0.0148). The non-parametric Kruskal-Wallis test followed by Dunn’s *post hoc* test was used for statistical analysis of wet weight data. The wet weight of isolated *E. multilocularis* cysts was lower in the PAB40 [0.26(IQR:0.20 ± 0.50) g, *p* < 0.01] groups than in the Untreated group [5.11(IQR:2.76–5.58) g]. In addition, the wet weight of cysts in the ABZ group [0.49(IQR:0.31–3.05) g] was lower than that in the Untreated group [5.11(IQR:2.76–5.58) g] (*p* < 0.05), but not significantly different compared with the PAB 10 and PAB 40 groups. Further HE staining was used to detect pathological changes in the cyst tissues of mice treated with PAB and ABZ. HE staining revealed the presence of distinct protoscoleces in the Untreated group, whereas protoscoleces were absent in both ABZ and PAB treated groups (Figure 5E). PAS staining showed strong positive reaction in the laminated layers of the cysts, but the laminated layers in the ABZ and PAB treatment groups were shed (Figure 5F).

### 3.8 Collagen deposition in the host tissue surrounding metacestodes

**Figure 6:**
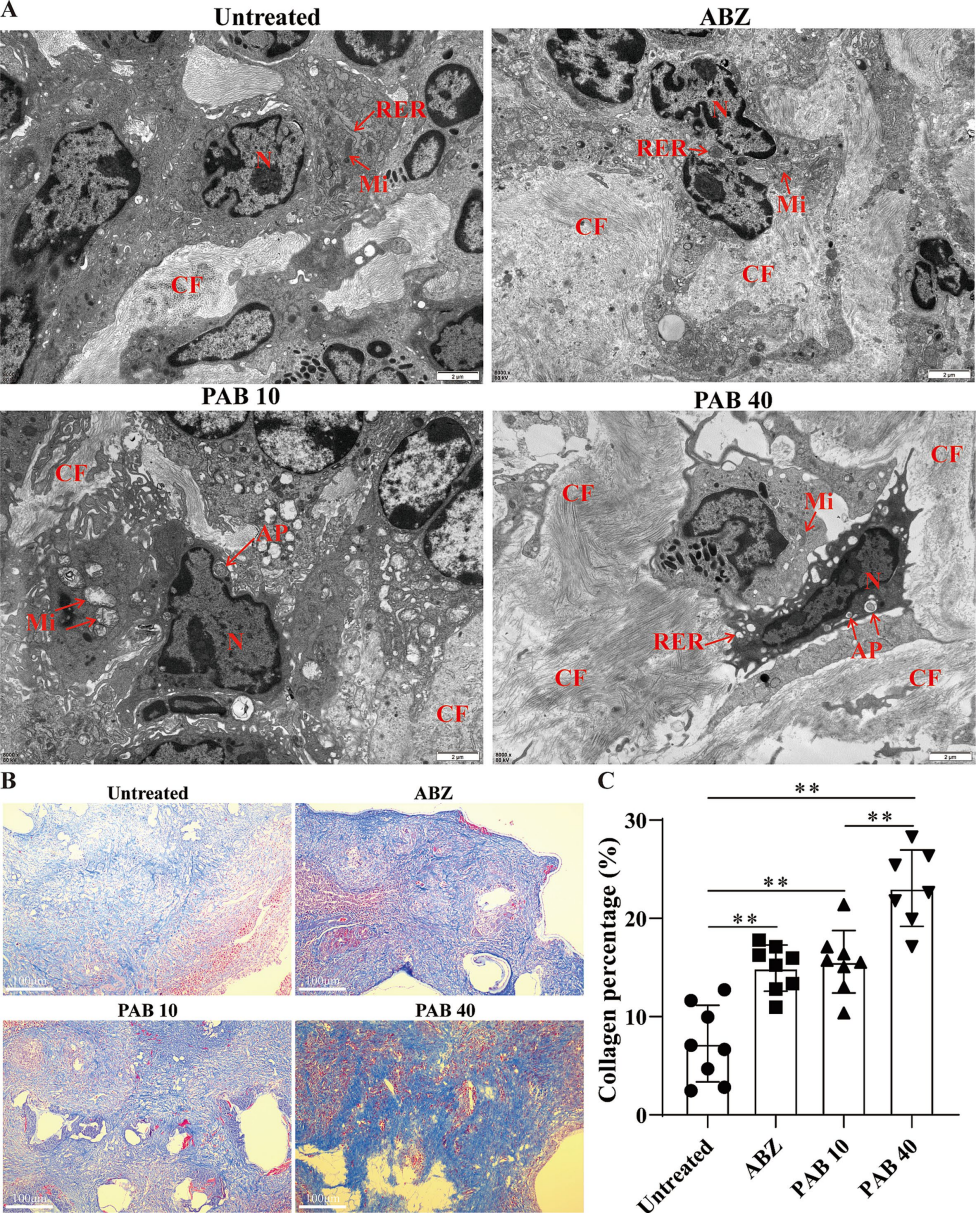
Effect of PAB on fibrosis in the host tissue surrounding the metacestodes. **(A)** TEM observation of host tissue surrounding metacestodes. N, nucleus; AP, apoptotic bodies; CF, collagen fiber; RER, rough endoplasmic reticulum; Mi, mitochondria. Scale bar = 2 μm. **(B)** Masson staining showed collagen fibers in the host tissue surrounding the metacestodes. Collagen fibers appear blue after staining. Scale bar = 100 μm. **(C)** Percentage of collagen fibers after PAB intervention. Data are presented as mean ± SD obtained from seven independent experiments. Statistical comparisons were performed using a Tukey’s post hoc test. ** Indicates *p* < 0.01.

The ultrastructural changes in the host tissue surrounding the metacestodes were observed via TEM (Figure 6A). In the Untreated group, abundant mitochondria and a rough endoplasmic reticulum were observed. In the ABZ group, the rough endoplasmic reticulum expanded and more collagen fibers were deposited in the intercellular space. In the PAB10 group, apoptotic bodies and collagen fibers were deposited in the intercellular substance. In the PAB40 group, apoptotic bodies appeared and collagen fibers were deposited in the intercellular substance. To further assess collagen fiber deposition, Masson’s trichrome staining was performed (Figure 6B). Masson staining confirmed that collagen fiber deposition in the host tissue surrounding the metacestodes was significantly increased in the PAB and ABZ treated groups compared to the control group (all *p* < 0.01) (Figure 6C). Furthermore, the PA40 group exhibited enhanced collagen synthesis compared to the ABZ group (*p* < 0.01).

### 3.9 PAB promotes apoptosis in host tissue surrounding metacestodes

**Figure 7:**
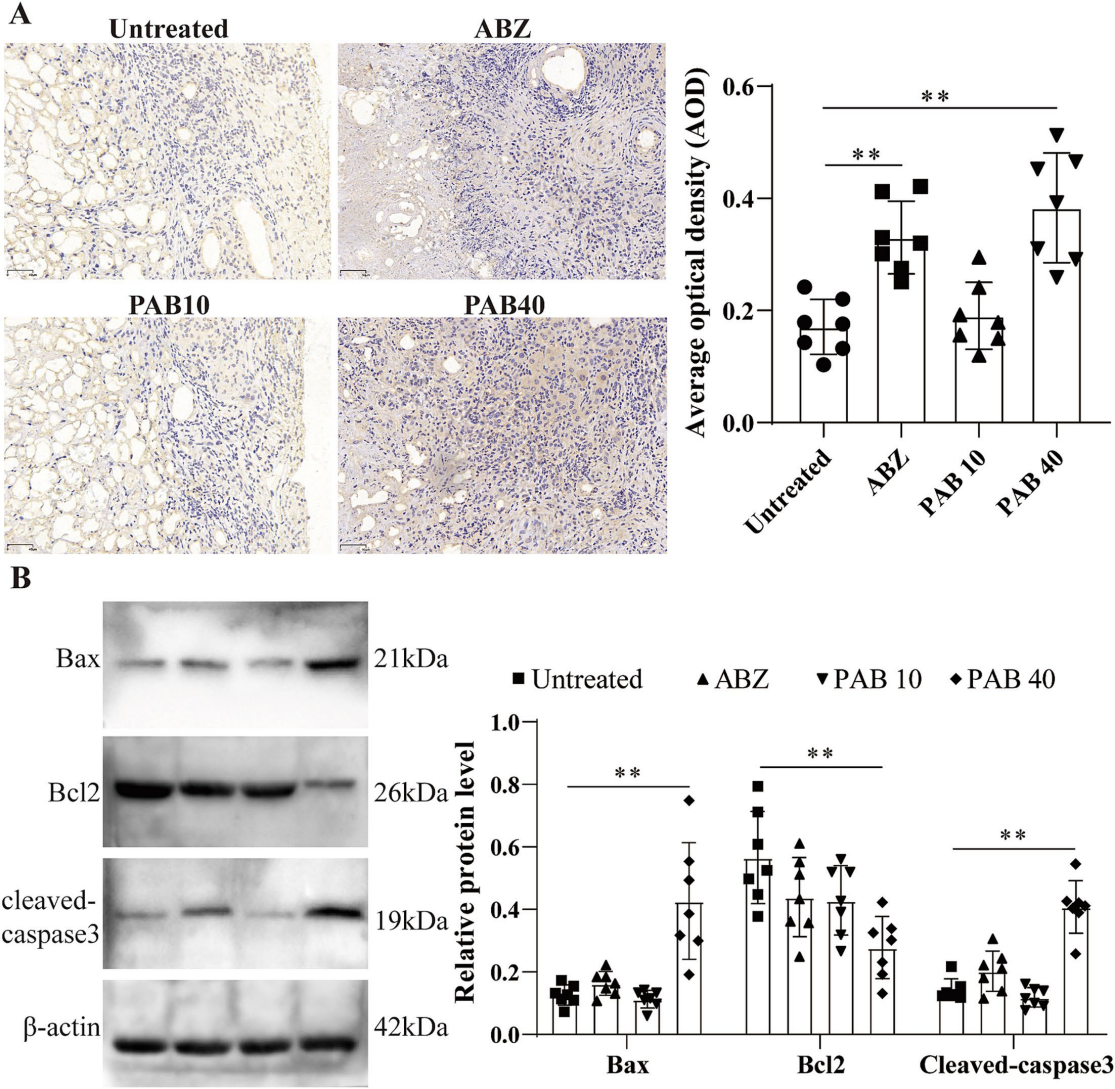
PAB promotes apoptosis in host tissue surrounding metacestodes. **(A)** Immunohistochemical detection of cleaved caspase 3 protein expression in host tissue surrounding metacestodes. Scale bar = 50 μm. Data are presented as mean ± SD obtained from seven independent experiments. Statistical comparisons were performed using a Tukey’s post hoc test. **(B)** Western blot was used to detect the expression levels of apoptosis-related proteins in the host tissue surrounding the metacestodes. Data are presented as mean ± SD obtained from seven independent experiments. Statistical comparisons were performed using a Tukey’s post hoc test. ** Indicates *p* < 0.01.

Based on the observation of apoptotic bodies in metacestode tissues, we further examined the expression patterns of key apoptosis-associated proteins in response to PAB administration. Immunohistochemistry was used to analyse cleaved caspase3 protein levels in host tissue surrounding metacestodes (Figure 7A). The results showed that the AOD was significantly higher in the ABZ (0.33 ± 0.06) and PAB40 (0.38 ± 0.10) groups compared to the Untreated group (0.17 ± 0.05) (all *p* < 0.05). To further confirm these immunohistochemical findings at the protein level, Western blot analysis was performed. Western blot analysis showed increased cleaved-caspase3 and bax, but decreased Bcl2 protein levels in the PAB40 group compared to the Untreated group in the host tissue surrounding the metacestodes (all *p* < 0.01, Figure 7B).

### 3.10 PAB regulates lymphocyte subsets in host tissue surrounding metacestodes

**Figure 8:**
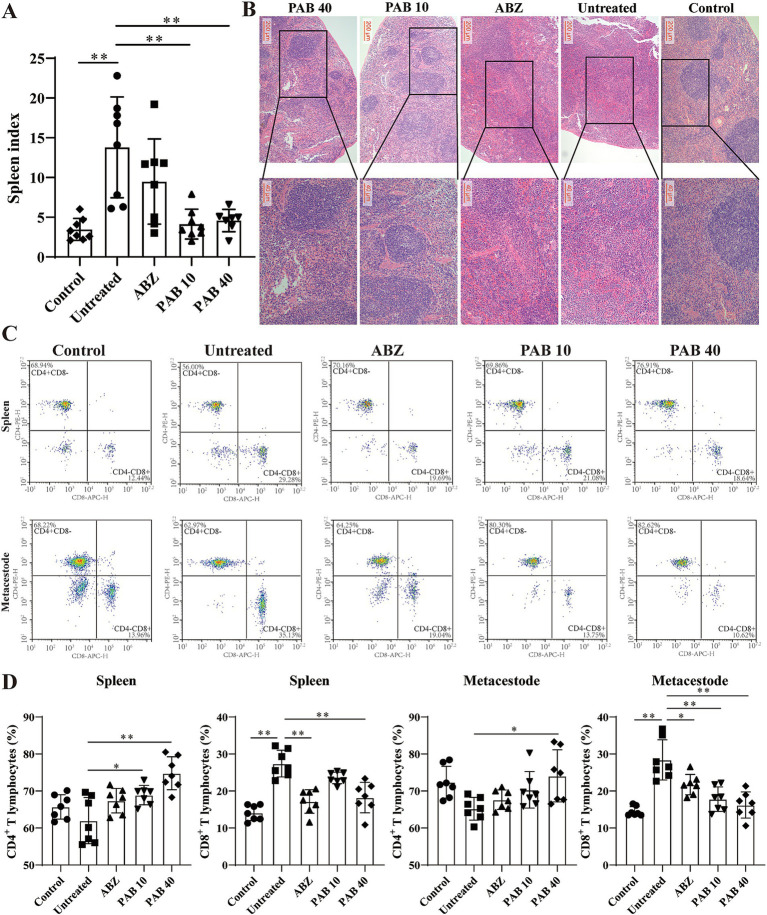
Variations in lymphocyte subsets in metacestodes and spleen. **(A)** Spleen indexes of mice treated with PAB. Data are presented as mean ± SD obtained from seven or eight independent experiments. Statistical comparisons were performed using a Tukey’s post hoc test. **(B)** Pathological changes in spleen were observed after HE staining. **(C)** CD3^+^ CD4^+^ T and CD3^+^ CD8^+^ T cells were detected via flow cytometry after staining with V450-CD3, APC-CD4, and PE-CD8 in the host tissue surrounding the metacestodes and spleen. Data are presented as mean ± SD obtained from seven independent experiments. Statistical comparisons were performed using a Tukey’s post hoc test. **(D)** Quantitative analysis of CD3^+^ CD4^+^ T and CD3^+^ CD8^+^ T cells in host tissue surrounding metacestodes and spleen. Statistical comparisons were performed using a Tukey’s post hoc test. * Indicates *p* < 0.05. ** Indicates *p* < 0.01.

Splenomegaly (enlargement of the spleen) is an important manifestation of inflammation induced by helminth infection (25). The decreased spleen weight in the mice after PAB treatment suggests that PAB may regulate the development of splenomegaly (Figure 8A). HE staining of the spleen in the PAB treatment group revealed well-preserved white and red pulp structures. The white pulp appeared intact and well-demarcated from the red pulp, similar to the control group (Figure 8B). Variations in lymphocyte subsets were evaluated to determine the immunoregulatory status and immune level of the host tissue surrounding the metacestodes and spleen. There was no significant change in the percentage of CD4^+^ T cells (Untreated: 62.00 ± 6.24% vs. Control: 65.72 ± 3.30%, *p* > 0.05) and an increase in the percentage of CD8^+^ T cells (Untreated: 27.42 ± 3.62% vs. Control: 14.10 ± 2.09%, *p* < 0.05) in the spleen in the Untreated group compared to the Control group. In contrast, after PAB treatment, there was an increase in the percentage of CD4^+^ T cells (PAB10: 68.90 ± 2.62% vs. Untreated: 62.00 ± 6.24%, *p* < 0.05; PAB40: 74.81 ± 4.45% vs. Untreated: 62.00 ± 6.24%, *p* < 0.01) and a decrease in the percentage of CD8^+^ T cells (PAB40:18.22 ± 4.12% vs. Untreated: 27.42 ± 3.62%, *p* < 0.01) in the spleens of the PAB10 and PAB40 groups compared with the Untreated group (Figures 8C,D). In addition, similar lymphocyte subpopulation manifestations were observed in the metacestode tissue as in the spleen (Figures 8C,D). There was no significant change in the percentage of CD4^+^ T cells (Untreated: 65.23 ± 3.06% vs. Control: 72.39 ± 4.29%; *p* > 0.05) and an increase in the percentage of CD8^+^ T cells (Untreated: 28.41 ± 5.42% vs. Control: 14.49 ± 1.28%, *p* < 0.01) in the metacestode tissue of the Untreated group compared to the Control group. Whereas, after PAB treatment, there was an increase in percentage of CD4^+^ T cells (PAB40: 74.09 ± 7.05% vs. Untreated: 65.23 ± 3.06%, *p* < 0.05) in metacestode tissues in the PAB40 group and a decrease in percentage of CD8^+^ T cells (PAB10: 17.81 ± 3.31%, PAB40: 16.22 ± 3.52% vs. Untreated: 28.41 ± 5.42%, all *p* < 0.01) in the PAB10 and PAB40 groups compared to the Untreated group. These results suggest that PAB treatment modulates the proportions of CD4^+^ and CD8^+^ T lymphocytes in both spleen and metacestode tissues, indicating its potential immunomodulatory effects.

### 3.11 PAB regulates Th1, Th2, Th17, and Treg balance in host tissue surrounding the metacestodes

**Figure 9:**
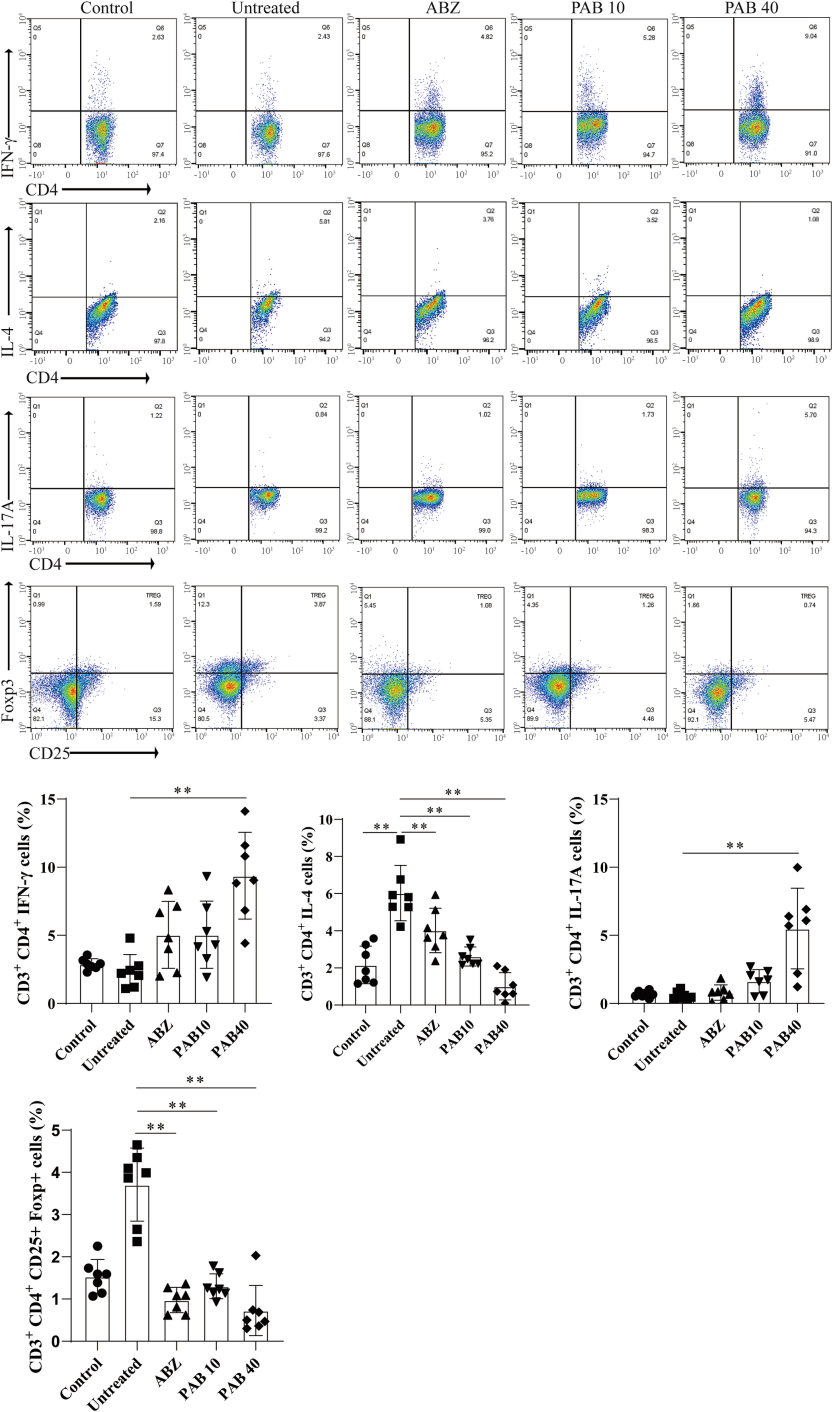
The percentages of Th1 (CD3e^+^CD4^+^IFN-*γ*^+^), Th2 (CD3e^+^CD4^+^IL-4^+^), and Th17 (CD3e^+^CD4^+^IL-17A^+^) lymphocytes in the host tissue surrounding the metacestodes and spleen were analyzed via flow cytometry, and the results of the statistical analysis are shown. Data are presented as mean ± SD obtained from seven independent experiments. Statistical comparisons were performed using a Tukey’s post hoc test. ** Indicates *p* < 0.01.

**Figure 10:**
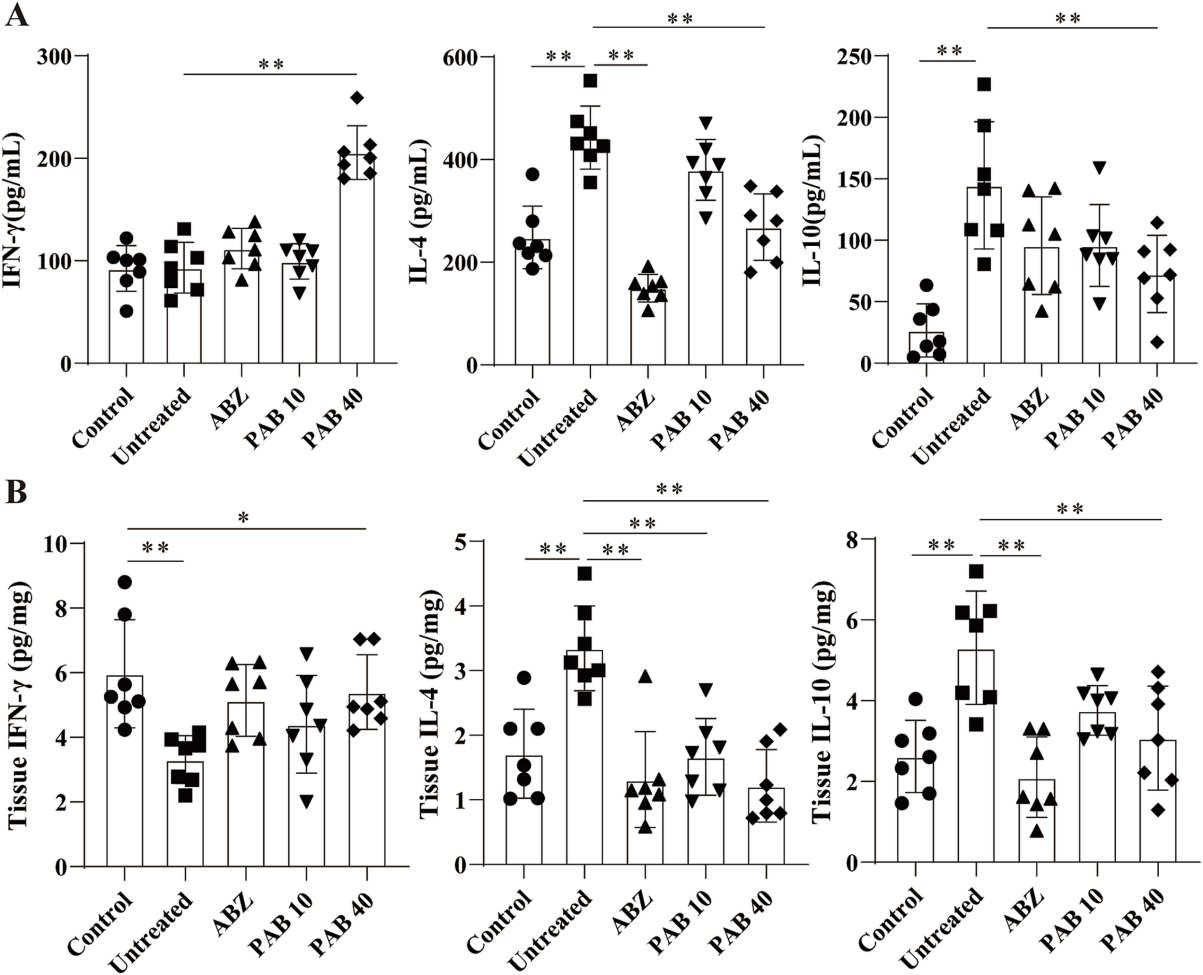
The levels of IL-4, IFN-γ, and IL-10 cytokines after PAB treatment. **(A)** Serum levels of IL-4, IFN-γ and IL-10. Data are presented as mean ± SD obtained from seven independent experiments. Statistical comparisons were performed using a Tukey’s post hoc test. **(B)** IL-4, IFN-γ and IL-10 levels in the host tissue surrounding the metacestodes. Data are presented as mean ± SD obtained from seven independent experiments. Statistical comparisons were performed using a Tukey’s post hoc test. * Indicates *p* < 0.05. ** Indicates *p* < 0.01.

Th2 cytokines are related to persistent hepatic fibrosis caused by helminth infection (26), and Th17 can promote the proliferation and activation of stellate cells (27). We further evaluated whether Th cells participated in the regulation of fibrosis in the host tissue surrounding the metacestodes after PAB treatment. The percentages of Th1, Th2, Th17 and Treg cells in the spleen and host tissue surrounding the metacestodes were detected through flow cytometry (Figure 9). We observed that PAB treatment upregulated CD4^+^ IFN-*γ*^+^ (Th1; PAB40: 9.37 ± 3.18% vs. Untreated: 2.36 ± 1.24%, *p* < 0.01) and CD4^+^ IL-17A^+^ (Th17; PAB40: 5.50 ± 2.95% vs. Untreated: 0.58 ± 0.29%, *p* < 0.01) cells, while downregulating CD4^+^ IL-4^+^ (Th2; PAB10: 2.62 ± 0.51%, PAB40: 1.01 ± 0.74% vs. Untreated: 6.03 ± 1.50%, all *p* < 0.01) cells compared to the Untreated group in the metacestode tissues. In addition, the percentage of CD4^+^ CD25^+^ Foxp3^+^ cells (Treg) in PAB-treated mice (PAB10: 1.30 ± 0.30%, PAB40, 0.64 ± 0.40%) was lower than that in the Untreated group (3.71 ± 0.87%) (all *p* < 0.01). Furthermore, serum levels of IFN-γ increased (PAB40: 205.60 ± 26.19 vs. Untreated: 93.30 ± 24.64 pg./mL, *p* < 0.01), while IL-4 (ABZ: 149.5 ± 26.69 pg./mL, PAB40: 268.50 ± 64.72 pg./mL vs. Untreated: 442.80 ± 61.35 pg./mL, all *p* < 0.01) and IL-10 (PAB40: 72.68 ± 31.46 pg./mL vs. Untreated: 144.7 ± 51.59 pg./mL, *p* < 0.01) levels decreased after PAB treatment compared to the Untreated group (Figure 10A). Moreover, IL-10 (ABZ: 2.11 ± 1.01 pg./mg, PAB40: 3.08 ± 1.29 pg./mL vs. Untreated: 5.31 ± 1.40 pg./mg, all *p* < 0.01) and IL-4 (ABZ: 1.31 ± 0.74 pg./mg, PAB10: 1.67 ± 0.59 pg./mg, PAB40: 1.22 ± 0.56 pg./mg vs. Untreated: 3.45 ± 0.66 pg./mg, all *p* < 0.01) levels were downregulated and IFN-γ (PAB40: 5.40 ± 1.16 pg./mg vs. Untreated: 3.31 ± 0.74 pg./mg, *p* < 0.01) level was upregulated in the metacestode tissues after PAB treatment (Figure 10B).

### 3.12 Regulatory effects of PAB on the expression levels of matrix metalloproteinases

**Figure 11:**
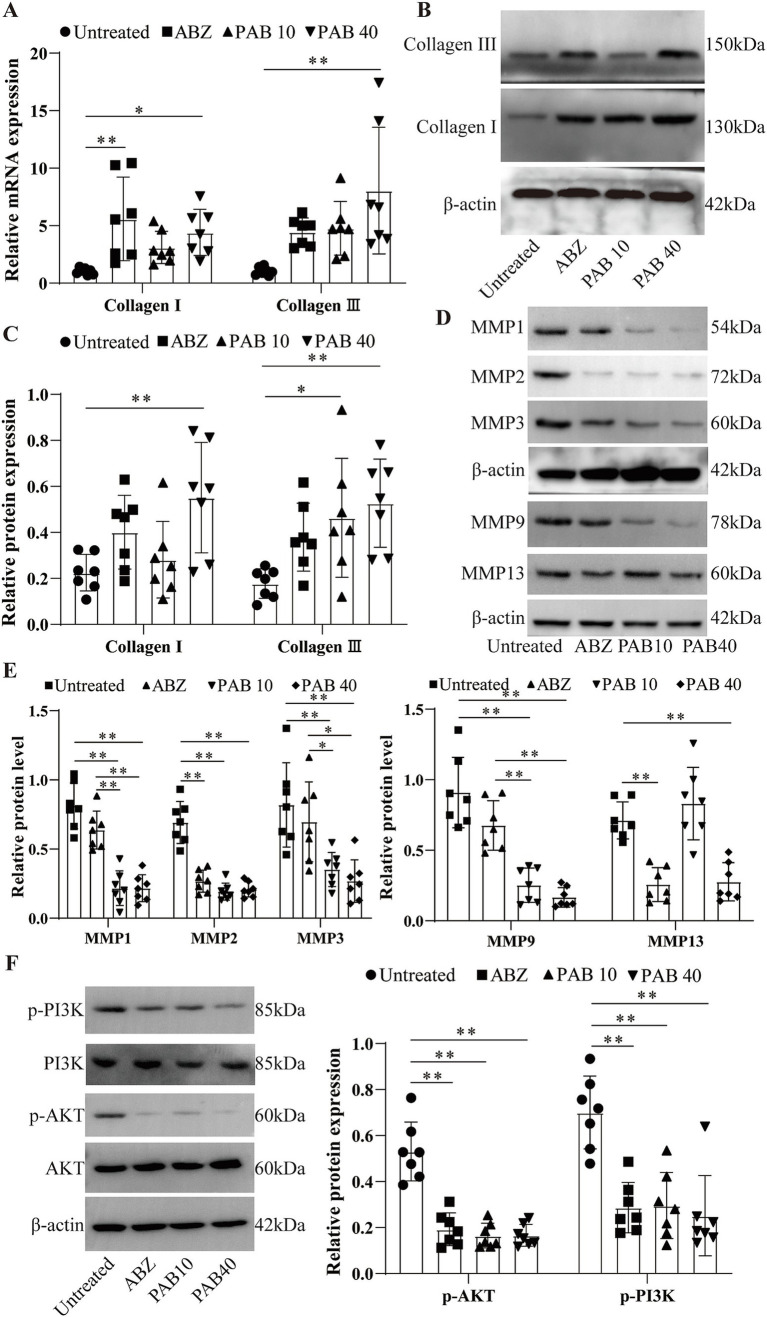
PAB promotes collagen synthesis in the host tissue surrounding the metacestodes. **(A)** Expression levels of collagen I and collagen III in the host tissue surrounding the metacestodes were analyzed via RT-qPCR. Data are presented as mean ± SD obtained from seven independent experiments. Statistical comparisons were performed using a Tukey’s post hoc test. **(B,C)** Western blot was used to detect the levels of collagen I and collagen III proteins. Data are presented as mean ± SD obtained from seven independent experiments. Statistical comparisons were performed using a Tukey’s post hoc test. **(D,E)** Western blot was used to detect the protein levels of MMP1, MMP2, MMP3, MMP9, and MMP13. Data are presented as mean ± SD obtained from seven independent experiments. Statistical comparisons were performed using a Tukey’s post hoc test. **(F)** Western blot for detection of PI3K/AKT protein levels in the host tissue surrounding the metacestodes. Data are presented as mean ± SD obtained from seven independent experiments. Statistical comparisons were performed using a Tukey’s post hoc test. * Indicates *p* < 0.05. ** Indicates *p* < 0.01.

Th1/Th2 cytokines tightly regulate MMPs through complex molecular mechanisms (28). MMPs play a key role in collagen degradation, so we also investigated the effects of PAB on MMPs production in the host tissue surrounding the metacestodes. After PAB treatment, collagen I and collagen III genes were upregulated 4.41-fold and 8.05-fold, respectively, in PAB40 group compared to Untreated group (*p* < 0.05; *p* < 0.01) (Figure 11A). Figures 11A,B show the results of Western blot analysis of collagen after PAB treatment. Compared to the Untreated group, collagen I levels were upregulated 2.45-fold in the PAB40 group (*p* < 0.01), while collagen III levels were upregulated 2.61-fold and 2.91-fold in the PAB10 and PAB40 groups, respectively (*p* < 0.05; *p* < 0.01). In addition, Western blot detected MMPs protein levels (Figures 11D,E). PAB treatment resulted in lower MMP1, MMP2, MMP3, MMP9, and MMP13 protein levels compared to the Untreated group. The most common signalling pathway upstream of MMPs is the PI3K/Akt signalling pathway, which regulates collagen production (29). Moreover, PAB can inhibit tumor growth and metastasis by targeting the PI3K/AKT pathway (30). Consequently, we further assessed the levels of PI3K/AKT signalling pathway proteins in the host tissue surrounding the metacestodes (Figure 11F). Compared to the untreated group, treatment with 10 mg/kg and 40 mg/kg PAB resulted in a 0.32-fold and 0.31-fold decrease in phosphorylated PI3K levels, and a 0.42-fold and 0.36-fold decrease in phosphorylated AKT levels, respectively (all *p* < 0.01). These findings demonstrate that PAB enhances collagen accumulation in the host tissues surrounding the metacestodes by downregulating MMPs level through inhibition of the PI3K/AKT signaling pathway.

## 4 Discussion

Alveolar echinococcosis (AE), a chronic zoonotic disease caused by the metacestode stage of *E. multilocularis*, poses a significant threat to public health, particularly in regions such as Central Asia, the Tibetan Plateau, and certain parts of Europe and North America (8). The current standard of care for AE relies on chemotherapy with albendazole and mebendazole, which are parasitostatic rather than parasiticidal (31). In addition, resistance to several commonly used antiparasitic drugs (such as benzimidazoles, ivermectin, and levoimidazoles) is increasing, and the emergence of resistance is associated with long-term use of the drugs, inadequate doses, and genetic variation of the parasites (32). Therefore, the search for new antiparasitic agents has increasingly turned to natural sources, including plants with traditional medicinal uses. The study by Aboubakr et al. on the protective effect of allicin and lycopene against methotrexate-induced testicular toxicity highlights the potential of natural compounds in mitigating the side effects of conventional drugs (33). These findings underscore the importance of exploring the therapeutic potential of herbal extracts and their active components in helminth control. PAB, a diterpene acid derived from the traditional Chinese medicine pseudolarix kaempferi gordon, demonstrated antifungal, anti-inflammatory and apoptotic effects (34). However, the underlying mechanisms of PAB’s action against *E. multilocularis* remain to be elucidated.

The present study demonstrated that PAB exhibited significant damaging effects on *E. multilocularis* metacestode vesicles. PAB treatment resulted in dose-dependent release of PGI in vesicles. PAB caused the vesicle structure to collapse and the germinal layer shrank from the laminated layer. PGI is prevalent in the metacestode vesicle fluid, facilitating nutrient absorption, and is only released into the medium when vesicles are mechanically damaged (35). The glycolytic enzyme PGI has been shown to stimulate the proliferation of parasite germinal layer cells and mammalian endothelial cells (36). The *E. multilocularis* germinal layer is a complex structure composed of glycogen storage cells, nerve cells, connective and muscle tissue cells, and undifferentiated stem cells, which is considered to be the reason for the high regenerative potential of *E. multilocularis* (18, 37). PAB inhibited the proliferation of germinal cells in a dose-dependent manner. By inhibiting germinal cell proliferation, PAB effectively targets the parasite’s ability to regenerate and expand, potentially halting disease progression (37). In addition, PAB exhibited dose- and concentration-dependent cytotoxicity against *E. multilocularis* protoscoleces, a finding supported by the suppressive effect of PAB on *E. multilocularis* involving the regulation of TGF-*β*1 signaling as described by Gao et al. (8). The observed destruction of the microvilli structure and the loss of rostellar hooks on the protoscolex surface, as evidenced by scanning electron microscopy, indicate that PAB disrupts the parasite’s ability to attach to and invade host tissues. This structural damage likely contributes to the observed reduction in protoscolex viability. These findings are consistent with those of Gao et al. (8), who also reported significant damage to the tegument and germinal layer of *E. multilocularis* protoscoleces after PAB treatment. Interestingly, both PAB and ABZSO treatment resulted in a reduction in glucose consumption by the protoscoleces and germinal cells. In the case of ABZSO, the primary mechanism of action involves inhibiting the polymerization of β-tubulin, which not only affects cell division, but also disrupts the transport of glucose within the parasite (38). However, further research is needed to clarify the precise mechanism by which PAB reduces glucose consumption in *E. multilocularis*. Overall, PAB exhibited higher toxicity against protoscoleces, metacestode vesicles, and germinal cells compared to human hepatocytes, HFFs cells, and HK2 cells.

In order to investigate the *in vivo* therapeutic effect of PAB, C57BL/6 mice were intraperitoneally infected with *E. multilocularis* metacestode vesicles. Notably, initial *in vivo* toxicity evaluations in C57BL/6 mice indicated that PAB treatment did not cause any changes in liver and kidney biochemical markers. Furthermore, no pathological alterations were observed in either the liver or kidneys. Previous studies have also confirmed that PAB is significantly less toxic than ABZ (8). The *in vivo* therapeutic effects of PAB were further confirmed on the basis of toxicity evaluation. Namely, PAB treatment resulted in a significant reduction in parasite burden compared with the Untreated mice. Moreover, compared to the ABZ treatment, the *in vivo* effect of PAB was comparable to that observed with ABZ after 4 weeks of administration (20), indicating the effective therapeutic activity of PAB for the treatment of *E. multilocularis* infection. The lesion histopathological examination showed that the germinal layer was obviously destroyed after PAB treatment. The germinal layer is crucial for their survival and development as it serves as the source of new cells and is responsible for the formation of protoscoleces (39). The observed destruction of this layer indicates that PAB directly targets and compromises the proliferative capacity of the metacestode. In addition, ultrastructural pathological changes in the surrounding tissues after PAB treatment showed increased apoptotic bodies and collagen deposition. Masson staining further confirmed the increase of collagen fibers after PAB treatment. Focal fibrosis as a natural barrier that can limit the growth and spread of the metacestode, thereby slowing down the progression of the disease (40). On the other hand, PAB treatment also resulted in increased levels of Bax and cleaved-caspase3, and decreased levels of Bcl-2 protein in the tissue surrounding the lesion. This result is consistent with previous studies showing that PAB induces G2/M phase arrest and apoptosis in several types of human cancer cell lines (41, 42). These findings highlight PAB’s potential dual action-direct parasiticidal effects and modulation of host tissue response, which may play a synergistic role in inhibiting parasite survival.

Previous studies have confirmed that patients with AE exhibit significant peripheral blood mononuclear cell proliferative responses to *E. multilocularis* antigens, suggesting that cellular immunity plays an important role in host–parasite interactions in AE (43). At the same time, echinococcus may achieve immune escape by producing antigens that cross-react with host T lymphocyte surface antigens (44). Recent studies have demonstrated that pseudolaric acid B can modulate the cytokine production and T-cell subpopulation functions (9). PAB treatment may alleviate parasite-induced splenomegaly, potentially by reducing parasite burden and thus the associated inflammatory processes. Further analysis of T lymphocyte subsets revealed that PAB treatment increased the proportion of CD4^+^ T cells and decreased the proportion of CD8^+^ T cells in the host tissue surrounding the metacestodes and the spleen. The CD8^+^ lymphocytes may contribute to the immunsuppression phenomenon investigated in AE (45). CD4^+^ T cells are crucial for coordinating effective anti-parasitic responses, which facilitate parasite clearance (46). This shift in CD4^+^ T and CD8^+^ T cells after PAB treatment is beneficial for infection control because it promotes the activation of immune cells directly involved in parasite destruction.

Naive CD4^+^ T cells can differentiate into at least four major lineages, Th1, Th2, Th17, and Treg cells, that participate in different types of immune responses (47). In the early stages of *E. multilocularis* infection, the host immune response may be characterised by a mixed Th1/Th2 profile, whereas in the middle to late stages of infection, the host immune response is mainly characterised by a Th2/Treg profile (48, 49). The observed shift in Th cell subpopulations and cytokine profiles further supports the immunomodulatory effects of PAB. Following PAB treatment, the increased Th1/Th17 and decreased Th2/Treg cell subpopulations in the metacestode tissues and spleen indicate a shift towards a Th1-dominant immune response. This shift is crucial for controlling *E. multilocularis* infection, as Th1 responses, characterized by the production of IFN-*γ*, are associated with parasite killing and clearance, while Th2 responses, characterized by IL-4 and IL-10 production, are often associated with parasite persistence and chronic infection (50). The upregulation of IFN-γ and the downregulation of IL-4 and IL-10 in the serum and periparasitic tissue following PAB treatment provide further evidence for this shift. Previous studies have shown that PAB’s amelioration of dermatitis severity is mediated by the reduction of proinflammatory cytokines and immune cell infiltration (34). In addition, *E. multilocularis* infection resulted in an increase of Treg cells in spleen and surrounding lesions, and Treg cells expressed more PD-1 and CTLA-4 to evade host immune response (48). PAB’s ability to shift the immune response towards a Th1 profile could potentially counteract this immune suppression. The reduction in Treg cells, which typically suppress immune responses, further suggests that PAB treatment may alleviate the immunosuppressive environment often associated with chronic *E. multilocularis* infection. This is in line with the early observations by Bresson-Hadni et al. suggesting that *E. multilocularis* may evade the host immune response by cross-reacting with T lymphocyte surface antigens (43).

The persistent infiltrative growth of *E. multilocularis* metacestodes in an intermediate host causes severe granulomatous inflammatory reactions and collagen accumulation around the parasites (51). The balance between different subpopulations of Th cells in AE plays a crucial role in the fibrosis process associated with the disease (52). The present study confirms that the increased deposition of collagen I and collagen III in the host tissue surrounding the metacestodes after PAB treatment may promote the fibrosis and calcification of the lesions and limit their growth. PAB can modulate the activity of fibroblasts, which are key cells responsible for collagen synthesis. By interacting with fibroblasts, PAB can potentially enhance the production of collagen, contributing to tissue repair and remodeling processes (53). Additionally, one study indicates that PAB can target CD147, a transmembrane glycoprotein that is known to regulate the expression of MMPs. By interacting with CD147, PAB may modulate the activity of MMPs, which are crucial for the degradation and remodeling of the extracellular matrix (54). We found that PAB could reduce the protein levels of MMP1, MMP2, MMP3, MMP9, and MMP13 in the host tissue surrounding the metacestodes. This change allows for the reduced degradation of collagen, which ultimately leads to extracellular matrix deposition. Therefore, the promotion of collagen production by PAB may be associated with attenuated MMP activity.

The PI3K/AKT signaling pathway contributes to tumorigenesis by promoting cell proliferation, regulating apoptotic and anti-apoptotic genes, and facilitating angiogenesis (55). PAB can inhibit tumor cell proliferation and migration and epithelial–mesenchymal transition process by inhibiting the PI3K/AKT signaling pathway (56). In this study, the p-PI3K and p-AKT protein expression was downregulated in the host tissue surrounding the metacestodes after PAB treatment. Furthermore, the activation of the PI3K/Akt signalling pathway is known to regulate MMPs and promote tumor invasion (57). In the context of cancer, inhibiting the PI3K/AKT pathway can reduce MMP expression and activity, thereby suppressing tumor cell migration and invasion (54). Therefore, PAB may exert an anti-echinococcosis effect by inhibiting the expression of MMPs through the modulation of the PI3K/AKT signaling pathway in the host tissue surrounding the metacestodes.

The study provides promising insights into the potential therapeutic effects of PAB against *E. multilocularis*, several limitations should be acknowledged. First, the precise molecular mechanisms by which PAB exerts its antiparasitic effects remain incompletely understood, particularly regarding its impact on glucose metabolism and the PI3K/AKT signaling pathway. Further research is needed to elucidate these pathways and confirm the direct targets of PAB within the parasite and host tissues. Additionally, the present study assessed cytotoxicity to parasite cells and assessed potential liver and kidney toxicity *in vivo*, but the potential effects of PAB on the reproductive system remain unexplored. Addressing these limitations will be crucial for advancing PAB as a viable therapeutic agent for AE.

While PAB shows great promise as a novel treatment for AE, it is crucial to consider the broader context of helminth control. The emergence of anthelmintic resistance, as highlighted by Qamar and Alkheraije in their review of Haemonchus contortus resistance, poses a significant threat to global efforts to control parasitic infections (32). The reliance on a limited number of anthelmintic drug classes has accelerated the development of resistance, emphasizing the urgent need for new therapeutic strategies. The parasitostatic nature of current AE treatments further exacerbates this concern, as long-term treatment can increase the selective pressure for resistance development. Vaccination offers a proactive approach, aiming to prime the host’s immune system to recognize and eliminate parasites before they establish significant infection. Significant progress has been made in vaccine research for fasciolosis, with nucleic acid vaccines and gene silencing methods showing 74 to 100% effectiveness (58). Recent advancements in vaccine development, as exemplified by the study by Zhou et al. on a multi-epitope vaccine against *E. multilocularis*, hold great promise for the future (59). These vaccines aim to enhance the host’s immune response by targeting multiple antigens, thereby improving efficacy and reducing the likelihood of immune evasion by the parasite. The cross-reactivity between parasitic antigens and T lymphocyte surface antigens, as discussed by Bresson-Hadni et al. (44), highlights the complexity of immune responses in AE and the potential for vaccines to overcome these challenges. In addition, herbal or natural compounds that usually exhibit less severe adverse drug reactions are widely used in anti-parasitic research (60). Their antiparasitic mechanisms may include the inhibition of protein synthesis, the induction of apoptosis, and interference with energy metabolism (60). Overall, the fight against helminth infections requires a multipronged approach. While PAB represents a significant step forward in AE treatment, continued research into new drugs, vaccines, and alternative therapies, including those derived from natural sources, is crucial to combatting the growing threat of anthelmintic resistance and achieving sustainable helminth control. The future of helminth control lies in a comprehensive strategy that integrates these diverse approaches to effectively address this global health challenge.

## 5 Conclusion

**Figure 12:**
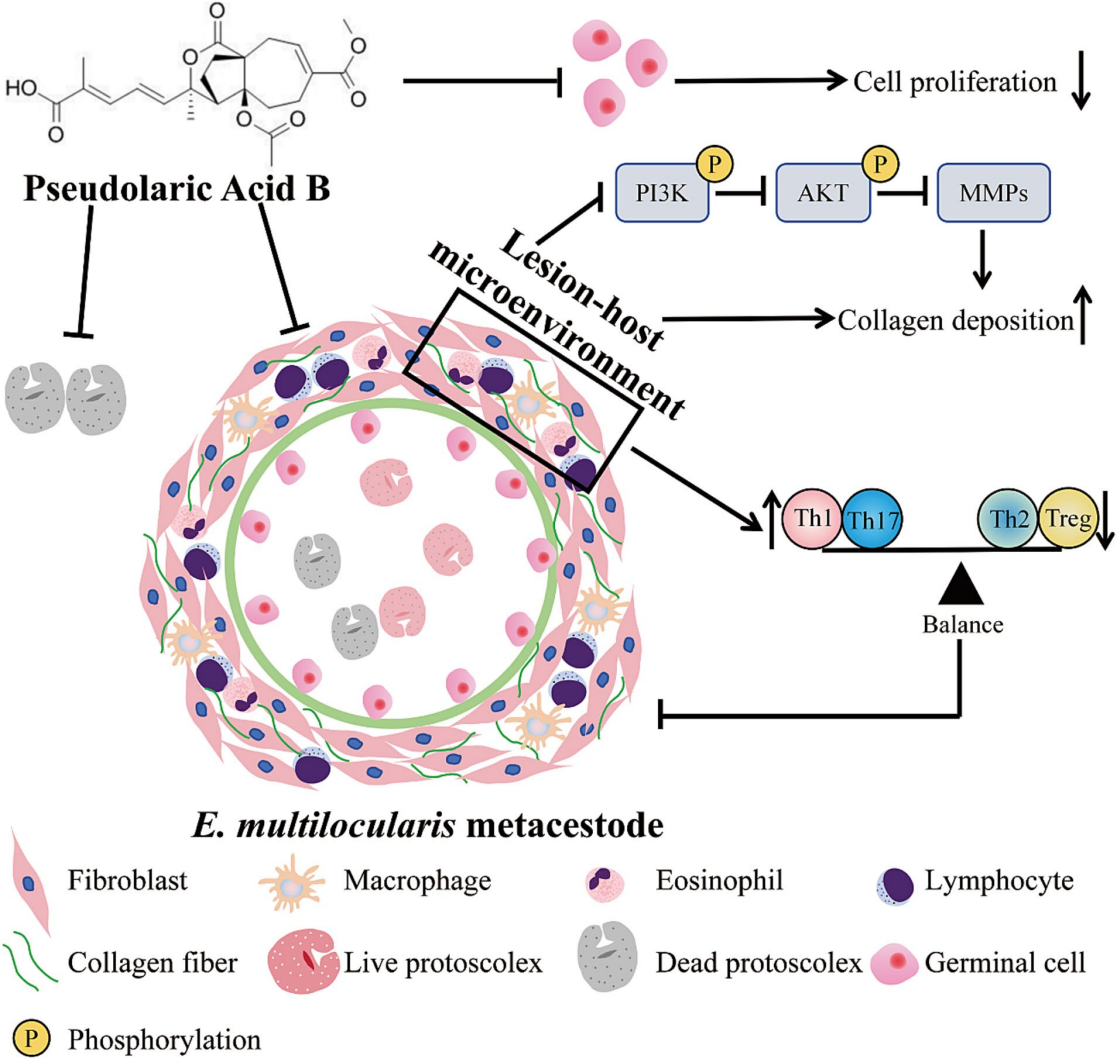
Schematic diagram of PAB used to target *E. multilocularis*.

In the present study, PAB demonstrated a potent killing effect on metacestode vesicles and protoscoleces at lower concentrations, and the toxicity of PAB towards mammalian cells was low. In addition, PAB exerted its anti-echinococcosis effect by modulating T lymphocyte function and promoting collagen deposition in the host tissue surrounding the metacestodes. These findings suggest that PAB not only directly targets the protoscoleces but also enhances the host’s immune response and potentially limits parasite growth and dissemination by reinforcing the physical barrier around the metacestodes. Figure 12 illustrates the proposed mechanistic hypothesis underlying our study. However, future research should focus on unraveling the targets of PAB’s action, exploring combination therapies, and gaining a comprehensive understanding of the host–parasite interactions in echinococcosis. These efforts will be crucial for the development of PAB as a promising lead compound against *E. multilocularis* and for advancing the treatment options for this challenging parasitic disease.

## Data Availability

The original contributions presented in the study are included in the article/Supplementary material, further inquiries can be directed to the corresponding authors.

## Footnotes

^1^ https://www.aatbio.com/tools/ic50-calculator

^2^ https://www.aatbio.com/tools/ec50-calculator
