# Supplementary material for: Efficacy and safety of pseudolaric acid B against Echinococcus multilocularis in vitro and in a murine infection model
Source: Front Med (Lausanne). 2025 Jan 29;12:1503472. doi: 10.3389/fmed.2025.1503472 (PMC11813755; doi:10.3389/fmed.2025.1503472)
Supplement: Supplementary file 1 [file Data_Sheet_1.pdf]

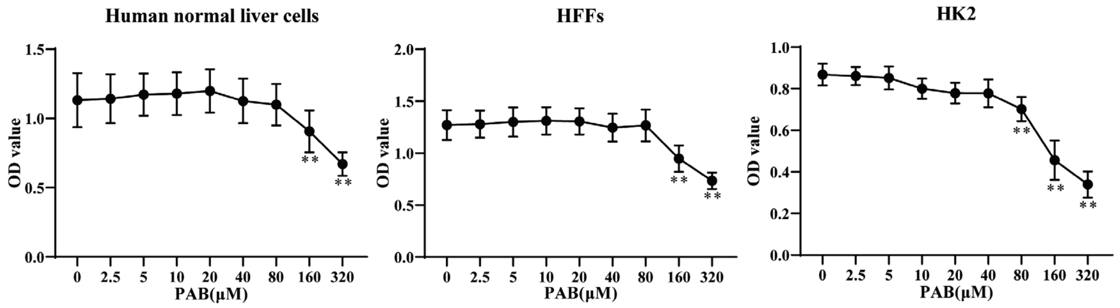

Figure S1. Cytotoxicity evaluation of PAB on mammalian cells. The activity of PAB after intervention in normal human hepatocytes, HFFs, and HK2 was determined via the CCK8 method. ** indicates $P < 0.01$.

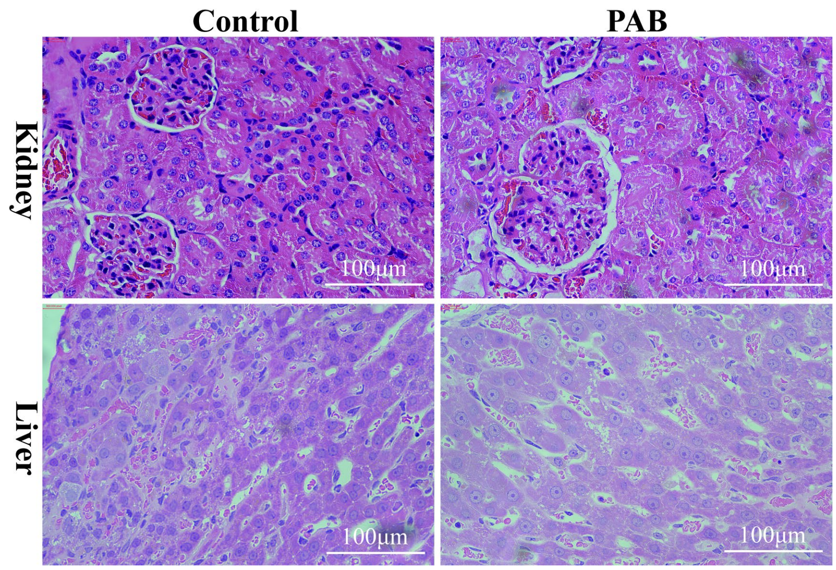

Figure S2. Histopathological examination of liver and kidneys in mice treated with PAB. Scale bar=100 μm.
